# Spectroscopic Determination of Acetylcholine (ACh): A Representative Review

**DOI:** 10.1007/s41061-023-00426-9

**Published:** 2023-05-11

**Authors:** Paweł Świt, Aleksandra Pollap, Joanna Orzeł

**Affiliations:** 1 Institute of Chemistry, Faculty of Science and Technology, University of Silesia in Katowice, 9 Szkolna Street, 40-006 Katowice, Poland; 2 Da Vinci’s International School, 4C Pilotów Street, Kraków, Poland

**Keywords:** Acetylcholine, Biological sample, Neurotransmitters, Sensors, Spectroscopy

## Abstract

Acetylcholine (ACh) is one of the most crucial neurotransmitters of the cholinergic system found in vertebrates and invertebrates and is responsible for many processes in living organisms. Disturbances in ACh transmission are closely related to dementia in Alzheimer’s and Parkinson’s disease. ACh in biological samples is most often determined using chromatographic techniques, radioenzymatic assays, enzyme-linked immunosorbent assay (ELISA), or potentiometric methods. An alternative way to detect and determine acetylcholine is applying spectroscopic techniques, due to low limits of detection and quantification, which is not possible with the methods mentioned above. In this review article, we described a detailed overview of different spectroscopic methods used to determine ACh with a collection of validation parameters as a perspective tool for routine analysis, especially in basic research on animal models on central nervous system. In addition, there is a discussion of examples of other biological materials from clinical and preclinical studies to give the whole spectrum of spectroscopic methods application. Descriptions of the developed chemical sensors, as well as the use of flow technology, were also presented. It is worth emphasizing the inclusion in the article of multi-component analysis referring to other neurotransmitters, as well as the description of the tested biological samples and extraction procedures. The motivation to use spectroscopic techniques to conduct this type of analysis and future perspectives in this field are briefly discussed.

**Graphical Abstract:**

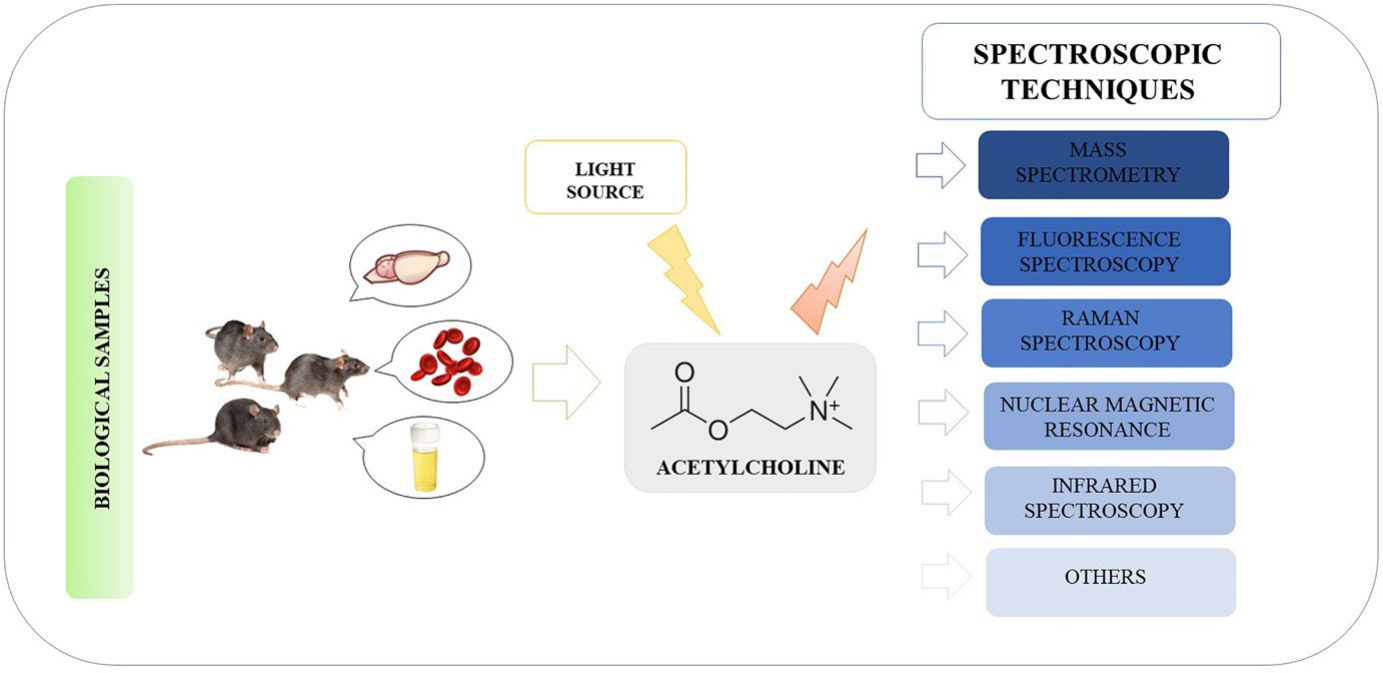

## Introduction

Neurotransmitters are chemical messengers responsible for carrying a multitude of signals between neurons (nerve cells) through synapses and from neurons to effector cells—muscle or glandular cells. Their action is manifested in perception, feeling, thinking, motor control, and cognition. Neurotransmitters are typically produced within nerve cells and may be released from them as a result of depolarization of the cell membrane and calcium-dependent exocytosis. Their role consists of transmitting that signal from one cell (called presynaptic) to another (called postsynaptic). The most common neurotransmitters are glutamate, γ-aminobutyric acid, acetylcholine, noradrenaline, dopamine, and serotonin [1–6].

One of the most crucial neurotransmitters found in vertebrates and invertebrates is acetylcholine (ACh), which constitutes the main transmitter of the cholinergic system (ChS) [7]. ACh was described at the beginning of the twentieth century by its discoverer Henry Hallett Dale. In 1921, Otto Loewi (an Austrian pharmacologist) named it and pointed out the existence of chemical conductivity [8–11]. The synthesis of ACh takes place in nerve terminals and is based on the reaction between acetyl coenzyme A (CoA) and choline (Ch), catalyzed by the enzyme choline acetyltransferase (ChAT). As a result of the ChAT activity, the acetyl group is transferred from the acetyl coenzyme and combined with Ch. This neurotransmitter is, therefore, an ester of acetic acid and Ch. After being released and evoking its action in the synapse, ACh molecules are hydrolyzed by acetylcholinesterase (AChE) to acetate and choline. Instead, the synthesized molecules that were not secreted from the presynaptic neuron into the synaptic cleft are stored in the granules. Furthermore, the presence of ChAT in neurons suggests that those cells use ACh as one of their transmitters [7, 11, 12].

The two major cholinergic projections in the brain can be distinguished: magnocellular basal forebrain ChS and brainstem ChS. The former is composed of the medial septal nucleus, the nucleus basalis of Meynert, the vertical and horizontal limbs of the diagonal band of Broca, and the substantia innominata. The basal forebrain ChS widely projects to different brain regions: neocortex, entorhinal cortices, hippocampus, basolateral amygdala, and olfactory bulb. Instead, the brainstem ChS, including the pedunculopontine nucleus and the laterodorsal pontine tegmental nucleus, primarily sends projections to thalamic structures and to basal forebrain regions [13, 14].

The ChS consists of two receptor families: the nicotinic receptors (nAChRs), belonging to the group of ionotropic receptors, and the muscarinic receptors (mAChRs), belonging to the group of metabotropic receptors. Both classes of these membrane-bound receptors are located in the central nervous system (CNS) and in the peripheral nervous system (PNS). The first type, nAChRs, are ion channels for Na^+^ and K^+^ ions, characterized by a fast signal transduction. Moreover, there are muscular (N1) and neural (N2) subtypes of nAChRs. Expression of nAChRs was mainly found in entorhinal, temporal, and primary motor cortices, hippocampus and thalamus, neuromuscular synapses, parasympathetic ganglia, and neuromuscular junctions, but also non-neuronal cells. The second receptor class, mAChRs, are G protein-coupled receptors (so-called GPCRs), and their activation leads to the formation of secondary messengers. Among five subtypes of mAChRs there are both excitatory (M1, M3, and M5) and inhibitory (M2 and M4). These receptors can be found in different tissues, mainly in the caudate nucleus and nucleus accumbens, the preganglionic and parasympathetic postganglionic neurons of the autonomic part of the PNS, smooth muscle, and endocrine glands. It is worth mentioning that within both ACh receptor families, several subclasses may be identified, both on the pre- as well as postsynaptic site [7, 11, 15–17].

ACh is a main effector in the autonomic nervous system [7]. In particular, in the autonomic nervous system ACh is a signaling molecule in the preganglionic sympathetic and parasympathetic neurons, and parasympathetic postganglionic fibers. Moreover, in the adrenal medulla ACh is used as a neurotransmitter at all organs innervated parasympathetically [18]. It plays a role as a transmitter at the sympathetically innervated piloerector muscle at the sweat glands and it forms terminals of neuromuscular synapses in the somatic system. Moreover, ACh may cause the contraction of muscle groups after binding to receptors located in PNS. Its action determines, among other things, secretion of saliva, milk, sweat ,or tears; ACh regulates heart contractions and blood pressure, is responsible for contracting intestinal muscles that results in moving the intestinal contents, controls urine release, causes erection, contracts skeletal muscles and those controlling near vision, causes adrenaline and noradrenaline release from adrenal glands, and through noradrenaline release from postganglionic fibers, ACh activates the sympathetic system [7, 8, 11, 18–21].

Apart from the important role of ACh in the PNS, ACh exhibits its effects on the CNS by changing neuronal excitability, impacting cellular and synaptic physiology, altering the presynaptic release of other neurotransmitters, and coordinating the firing of neurons [9, 22]. ACh being released from cholinergic neurons, that project to various brain regions, maintain the excitation–inhibition balance among neuronal circuits [7, 11, 12, 23]. In the CNS, ACh is responsible for processes such as arousal, attention, memory (long-term and working memory, memory formation, consolidation, and retrieval), and motivation. ACh is considered as a morphogen since it is found in the first moments of the ectodermal system development (neuronal plate) and is crucial for the differentiation of neural cells. Additionally, it provides communication between different CNS areas and switches network dynamics, causing behavioral transitions (e.g., from sleep to wakefulness, distraction to attention, learning, and recall). Its range includes primarily such structures as the hippocampus, which is mainly responsible for memory, and neurons of the tegmental nuclei and interbranch nuclei in the brain, which regulate vegetative activities, e.g., sleep. Moreover, the basal forebrain innervates the dense neocortex that coordinates higher levels of cognitive processes. Thus, the activity of the ChS is associated with both peripheral and central functions, being especially associated with the motor functions of muscles, with learning and memory, as well as with the greater organization of human consciousness [7, 8, 11, 18–21].

The importance of the ChS is supported by the fact that impairments in the cortical cholinergic innervation are closely associated with dementia of Alzheimer’s and Parkinson’s disease [18, 24]. It was proven that cholinergic neurons undergoing age-related moderate changes result in cholinergic hypofunctions and, thus, produce memory deficits and dementia [13]. Interestingly, ACh deficiency is also manifested by impaired rapid eye movement (REM) sleep, or its complete elimination, which results in a deterioration of memory and concentration. Likewise, disorders of the ChS can lead to many negative gastric symptoms, since it is a key regulator of gastrointestinal motility and pancreatic secretion [25].

**Fig. 1:**
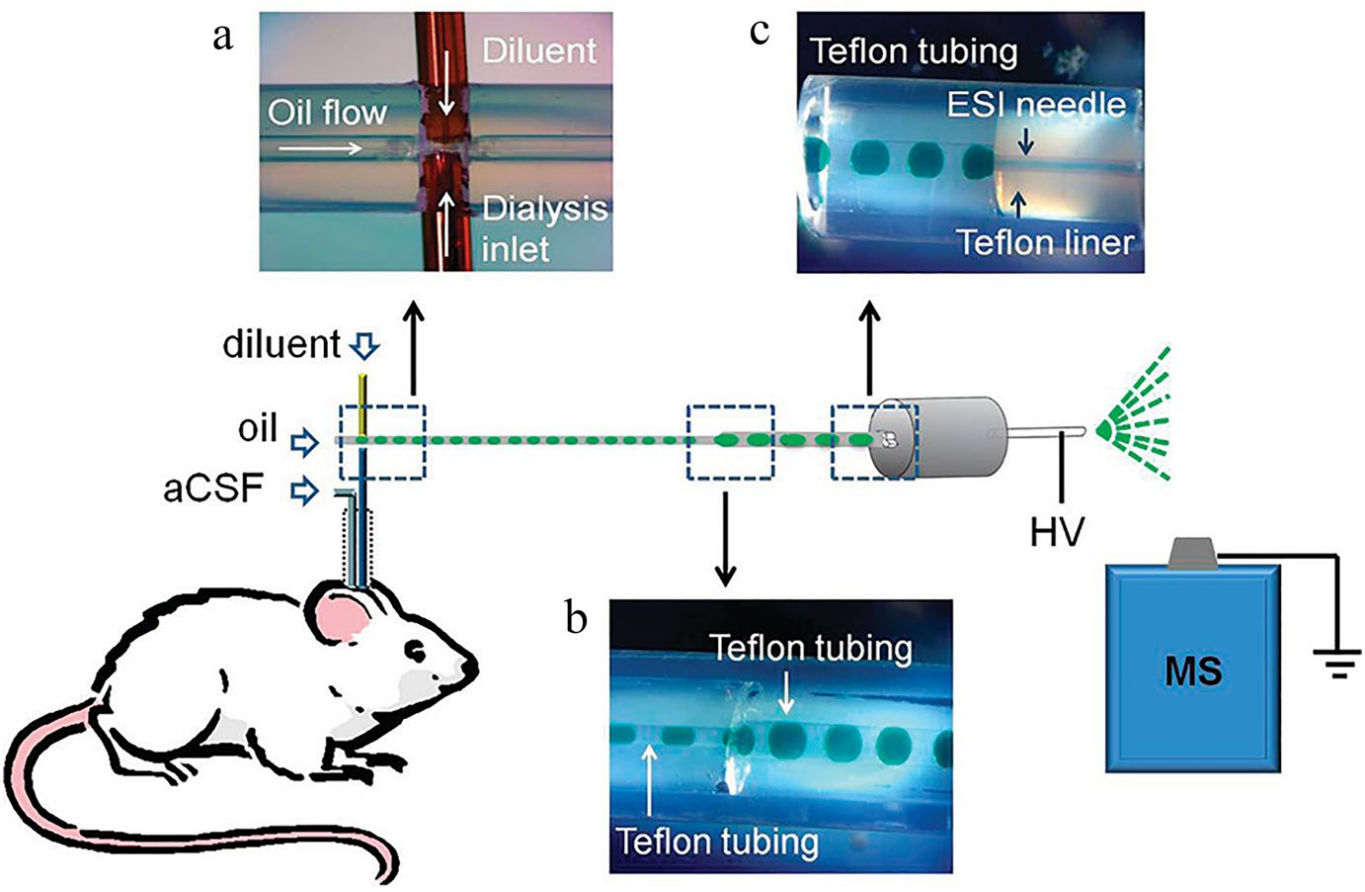
Scheme presenting the combination of microdialysis to segmented flow electrospray ionization mass spectrometry (ESI–MS): a—droplet generation device before it was sealed with epoxy, b—droplet coalescence connection, and c—liquid connection at ESI probe. Reprinted with permission from Ref. [28]. Copyright (2012) American Chemical Society

ACh is often detected and determined in biological samples, e.g., blood, serum, plasma, urine, or tissues after homogenization. An extremely interesting approach is microdialysis, despite it still not having great applicability, which is a result of the need to have advanced equipment. ACh in the biological samples of animals from CNS disease models is most often detected and determined in the cerebrospinal fluid (CSF) collected from various brain structures, most often by microdialysis. Microdialysis in freely moving laboratory animals is an analytical technique that allows dynamic monitoring of the concentration of a number of substances in living tissue (in vivo), including the release of neurotransmitters in the extracellular fluid, which is its great advantage. Firstly, a microdialysis probe, a small dialysis catheter with a semipermeable membrane, is inserted into the investigated brain region. Then, microdialysis samples obtained by perfusion with artificial extracellular fluid are collected [26, 27]. Figure 1 shows an example of a procedure that includes microdialysis [28]. This technique has been used to determine ACh using spectroscopic techniques in many literature reports.

The fundamental limitation in the study of this type of biological samples is a very complex sample matrix, low concentrations of the substances to be detected and determined, and small sample volumes (of the order of a few microliters). For this reason, it is necessary to use appropriate methods that will allow obtaining reliable information on the occurrence and concentration of this neurotransmitter in biological samples. These types of brain dialysates samples are analyzed using radioenzymatic assays, enzyme-linked immunosorbent assay (ELISA), or potentiometric methods, but also the following analytical techniques: high-performance liquid chromatography (HPLC) or gas chromatography (GC), with electrochemical (ED), fluorescence (FLD), or optical absorption detection in the ultraviolet (UV) range, as well as in combination with mass spectrometry (MS) [27].

Liquid chromatography in the HPLC-ED version is most often used for the determination of ACh [22, 29–40]. This method is based on the integration of several steps: (1) separation of ACh on a dedicated column (e.g., a micropores reversed-phase column) under ion evaporation, (2) on-line conversion (enzymatic) of ACh to hydrogen peroxide, and (3) electrochemical analyte detection on an electrode (e.g., a platinum electrode). This type of approach, with some modifications, such as the type of analytical column or the material from which the electrode is made, but still based on the principle presented above, is the most commonly used method for the determination of ACh in biological material [22, 29–40]. In recent years, a method for the determination of ACh in dialysates has been presented along with various methodological approaches to the calculation of the analytical result [29]. In this work, the occurrence of interference effects associated with a very complex matrix of samples was demonstrated, and the accuracy of the obtained results was assessed, together with an indication of the size of systematic errors made using traditional methods.

Alternative methods to detect and determine ACh are facilitated by spectroscopic techniques. Most spectroscopic techniques are fast and accurate compared with wet chemical methods, like ELISA, making them convenient for routine analysis. With these techniques, simultaneous studies of several parameters with a single measurement is possible, in comparison with tests using separation techniques with detection under one measurement conditions. Moreover, in some cases sample preparation for spectroscopic measurements is simple, fast, and cost effective. Unfortunately, in some cases it is necessary to use very advanced equipment and complicated procedures for sample preparation using antibodies compared with separation techniques. Most of the spectroscopic techniques can be considered as non-destructive, which makes it possible to take measurements using different methods on the same sample. Some techniques, such as surface-enhanced Raman spectroscopy (SERS), are characterized by very good limits of detection that allow for obtaining analytical signals even for single molecules. An up-and-coming technique is MS, which is characterized by high accuracy of mass determination, resolution up to several atomic mass units, and a wide range of applications. MS is an analytical technique classified as a spectroscopic method on the basis of the measurement of the ratio of mass to the electric charge of a given ion. In the case of ACh testing, MS spectrometry is most often coupled with chromatographic techniques [41]. In this aspect, the use of, for example, the HPLC technique with ED detection is a much cheaper solution, but it does not allow for such low detection limits as it does in SERS spectroscopy or MS. In addition, MS spectrometry can visualize the presence of ACh in different tissue areas, which is impossible with chromatographic methods. Each time when choosing the best research method, it is necessary to take into account what is the goal of conducted research and what kind of sample is to be analyzed. Due to the mentioned features of spectroscopic techniques, despite some limitations, their application is an extremely interesting prospective approach used to determine ACh.

**Fig. 2:**
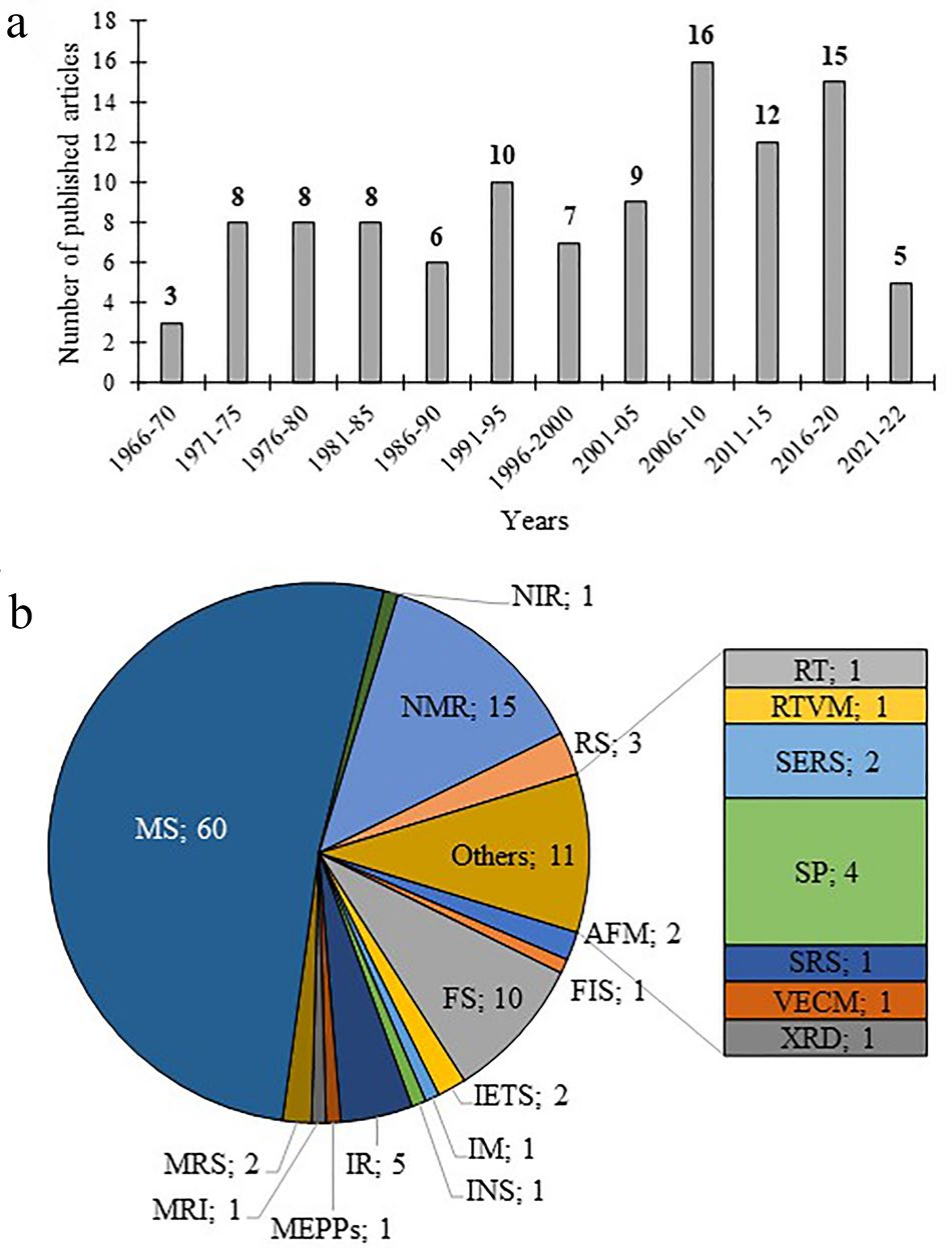
Graphs on the number of published articles and the types of spectroscopic techniques used. **a** Number of published articles on the detection and determination of ACh from 1966 to December 2022 based on the following databases: ScienceDirect, Scopus, Web of Science, Google Scholar, and PubMed. **b** A graph showing the number and type of spectroscopic techniques used, where: *AFM* atomic force microscopy, *FIS* Faradaic impedance spectroscopy, *FS* fluorescence spectroscopy, *IETS* inealestic electron tunneling spectroscopy, *IM* imaging, *INS* incoherent neutron scattering, *IR* infrared, *MEPPS* miniature end-plate potentials, *MRI* magnetic resonance imaging, *MRS* magnetic resonance spectroscopy, *MS* mass spectrometry, *NIR* near infrared, *NMR* nuclear magnetic resonance, *RT* radiometric techniques, *RS* Raman spectroscopy, *TRVM* real-time video microscopy, *SERS* surface enhanced Raman spectroscopy, *SP* spectrophotometry, *SRS* stimulated Raman spectroscopy, *VECM* video-enhanced contrast microscopy, *XRD* X-ray diffraction

This article presents a review of the available scientific literature on spectroscopic techniques used for the detection and determination of ACh, especially in animal models as an alternative to commonly used chromatographic techniques. Additionally, this article presents a discussion of examples of other biological materials (blood, plasma, serum, urine, and tissues) from animals, as well as clinical and preclinical studies to give the whole picture of the application of spectroscopic methods. The aim of the article is to show the last achievements in this area in historical reference dating back to the 1960s. In the literature review, the oldest articles were published in 1966, and the latest in December 2022. The following databases: ScienceDirect, Scopus, Web of Science, Google Scholar, and PubMed, were used to search for articles. In the mentioned period, 107 articles were found showing the detection and determination of ACh using spectroscopic techniques (Fig. 2a). Until 2015, the number of articles in the 5-year period remained constant, with an average of eight articles published. Since 2006, an almost two-fold increase in publications has also been observed in the 5-year period, so it can be concluded that a similar trend will occur in the years 2021–2025. The most common spectroscopic techniques are presented in Fig. 2b. The most significant number of reports, amounting to 60, concerns the use of MS, most often as a detection technique after the use of chromatographic separation. The second largest group consisted of reports on the use of the nuclear magnetic resonance (NMR) technique. However, these studies were mostly related to structural analysis. Fluorescence spectroscopy (FS) is the third most numerous group, and it was used mainly in the development of sensors allowing the detection of ACh. Techniques such as infrared (IR), Raman spectroscopy (RS), and spectrophotometry (SP) were mentioned between three and five times in published studies. Other techniques were used less frequently, appearing in the conducted research no more than two times. In the coming years, the SERS technique may prove to be an excellent tool for the determination of ACh in biological samples, which is associated with high detection capabilities (even a single molecule) and the stability of the recorded signal. This technique, together with fluorescence, may prove to be perfect mainly for developed sensors, also with the use of microfluidic systems. This article also presents a discussion of sensors for ACh-based research on the use of spectroscopic techniques.

## Spectroscopic Techniques for the Determination of Acetylcholine

**Fig. 3:**
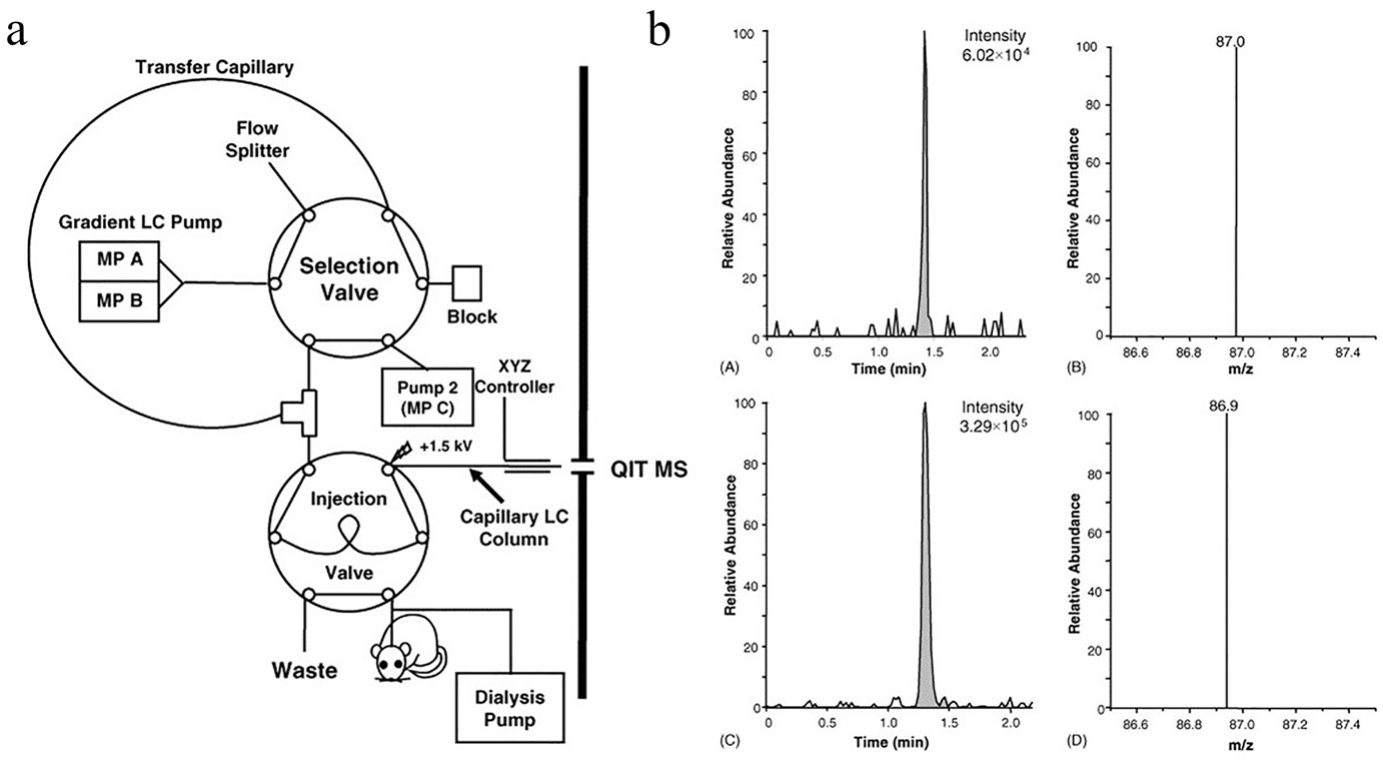
**a** Diagram of the cLC-MS system. Reprinted from Journal of Neuroscience Methods, 159/1, Holly M. Shackman, Minshan Shou, Nicholas A. Cellar, Christopher J. Watson, Robert T. Kennedy, Microdialysis coupled on-line to capillary liquid chromatography with tandem mass spectrometry for monitoring acetylcholine in vivo, 86–92, Copyright (2007), with permission from Elsevier. **b** Comparison of mass chromatograms for ACh in standard solution prepared in CSF and basal levels from the striatum of a ketamine anesthetized rat, where: **A** ACh standard at concentration 330 pM and **B** corresponding to selected reaction monitoring (SRM) MS/MS spectra, **c** basal ACh, and **D** corresponding to SRM MS/MS spectra. Reprinted from Journal of Neuroscience Methods, 159/1, Holly M. Shackman, Minshan Shou, Nicholas A. Cellar, Christopher J. Watson, Robert T. Kennedy, Microdialysis coupled on-line to capillary liquid chromatography with tandem mass spectrometry for monitoring acetylcholine in vivo, 86–92, Copyright (2007), with permission from Elsevier

**Fig. 4:**
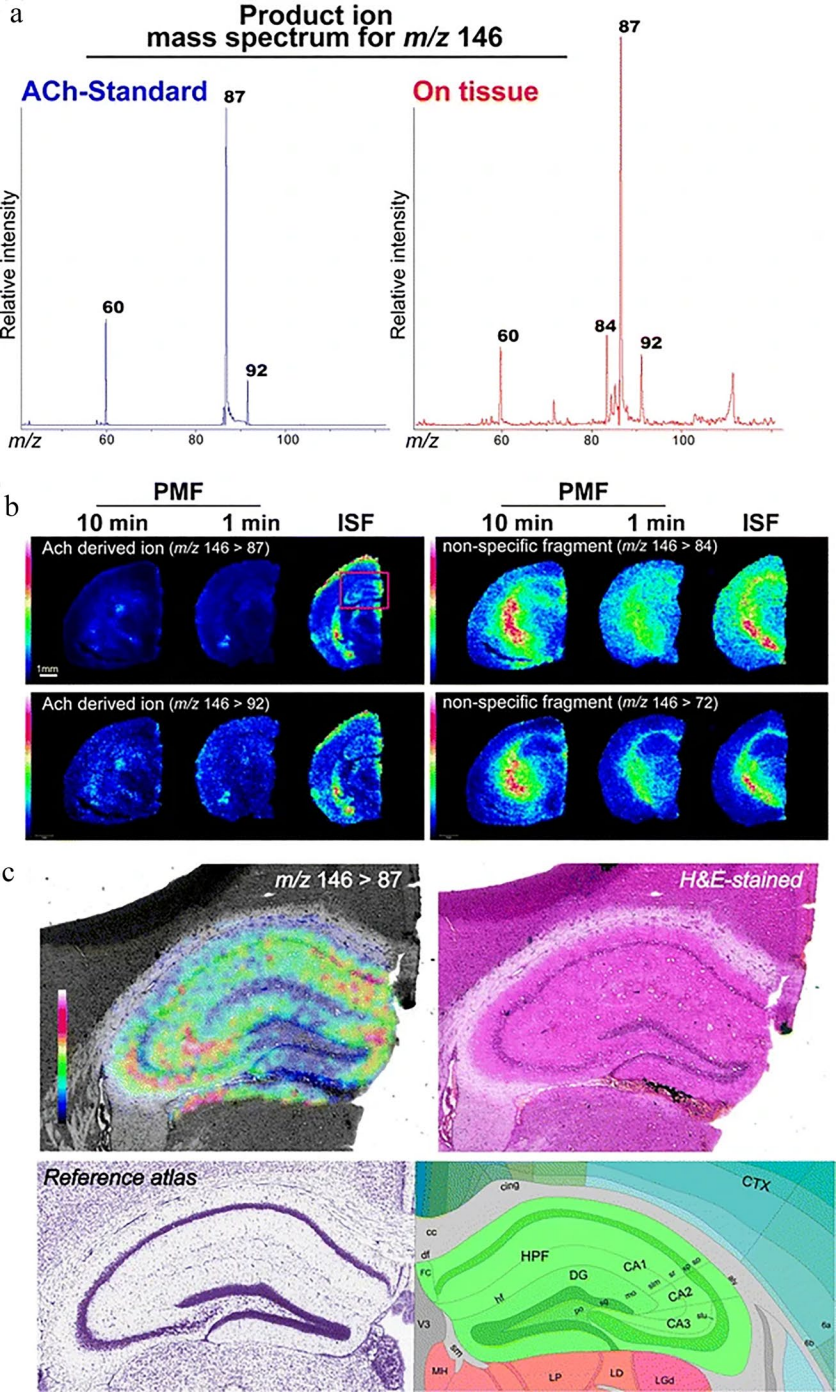
Exemplary figure presenting visualization of ACh distribution in CNS tissue sections by tandem imaging MS. **a** Mass spectrum received from ACh standard and on brain tissue, **b** images representing ACh and other ion distribution (three groups of mice treated with different animal fixation methods), and **c** image of ACh distribution in the hippocampus. The figure is from an open access article distributed under the terms of the Creative Commons CC BY license. Copyright © 2012. Springer, Nature

Any new analytical method should be well described according to the crucial sample preparation steps, measurement parameters, and analytical parameters characterizing its performance. A summary of selected basic parameters [e.g., linearity range, determination coefficient, analytical curve equation, limits of detection (LODs) and quantification (LOQs)] characterizing the performance of methods developed for ACh analysis with spectroscopic methods, reported in the available literature, is included in Table 1. It should be stressed that some research contains only shredded information of considered analytical performance. Thus, they are not included in the tabular form of the summary. However, the authors report chromatographic methods coupled with MS of nano- and picomole working ranges [42–47] and LODs at the picomole level [44–49] and femtomole level [50]. A number of other applications of MS spectrometry can be found in the literature [51, 52]. Figures 3 and 4 show the examples of tests using MS spectrometry [53, 54]. The lowest reported LOD for ACh, obtained using SERS, is at attomolar level (precisely 1 aM = 1 × 10^–18^ mol/L) [55].

**Table 1:** Summary of analytical performance parameters for detection of ACh with spectroscopic techniques

Spectroscopic technique	Sample	Linearity range	*R*^2^ (equation)	LOD/LOQ	References
MS (GC–MS)	Rat blood	0.5–4 pmol	0.9998	0.3 pmol/–	[89]
MS (capillary GC–MS)	Canine brain and blood	10–400 nmol/L	–	0.5 pmol/–	[90]
MS (thermospray LC–MS)	Mouse brain	0.030–30 nmol	0.9996 (*y* = 1.0048*x − *0.0166)	30 pmol/–	[91]
MS (GC–MS)	Rat brain	5–100 pmol	0.998	0.5–2.0 pmol/–	[92]
MS (LC–MS)	Rat brain	to 20 pmol	0.99989	1 pmol/–	[93]
SP	Synthetic	7.5–62.5 μmol/L	0.999	–/–	[94]
MS (GC–MS)	Rat brain	2.5–20 nmol	0.9996	2 pmol/–	[95]
MS (HPLC–MS)	Rat pheochromocytoma cell line (PC12)	1–100 pmol	– (*y* = 0.995*x* + 0.0075)	0.3 pmol/–	[96]
MS (LC–MS/MS)	Rat brain microdialysates	0.1–1000 nmol	0.9792	0.1 nmol/0.5 mol	[97]
MS (LC–APCI/MS/MS)	Rat corneal epithelium	6.84–1710 pm/L	0.998 (*y* = 2.65 × 10^−3^*x* − 8.22 × 10^–3^)	6.84 pm/L/20.52 pm/L	[98]
MS (LC–MS/MS)	Mice and rat brain microdialysates	0.1–50 nmol/L	0.999	0.02 nM/0.1 nmol/L	[99]
MS (LC–MS/MS)	Rat brain microdialysates	up to 5 nmol/L	–	0.1 nmol/L/–	[100]
MS (LC–APCI/MS/MS)	Rat brain microdialysates	0.15–73 nmol/L	0.986 (*y* = 0.0302*x* + 0.0004)	–/0.15 nmol/L	[101]
MS (LC–ESI/MS/MS)	Rat brain microdialysates	0.05–10 nmol/L	> 0.995	0.2 fmol/0.05 nmol/L	[102]
MS (LC–MS/MS)	Rat brain microdialysates	0.33–33 nmol/L	> 0.98	0.04 nmol/L/–	[53]
FLD	Chemical standard	0.05–100 µmol/L	–	0.5 nmol/L/–	[103]
MS (HILIC–MS/MS)	Human liver	6.84–1368 pm/L	> 0.999	1.37 pmol/L/4.10 pmol/L	[104]
MS (HILIC/ESI–MS/MS)	Rat microdialysate	0.025–50 nmol/L	0.9994	0.075 fmol/0.25 fmol	[105]
MS (HILIC–LC–MS/MS)	Cell lysate (SN56.B5.G4)	0.001–10 µmol/L	0.997 (*y* = 0.0099*x* + 0.0034)	0.3 nmol/L/1 nmol/L	[106]
MS (LC–ESI–M/MS)	Rat brain microdialysate	0.5–15 nmol/L	0.9944 (*y* = 2.528*x* + 0.302)	0.31 nmol/0.92 nmol	[107]
NIR–FLD	Synthetic	0.5–1 mmol/L	–	0.05 mmol/L/–	[108]
MS (MALDI–TOF MS)	Mouse brain cerebrospinal fluid	1–1000 nmol/L	0.9996	0.3 nmol/L/1 nmol/L	[109]
MS (UPLC–MS/MS)	Human plasma and urine	0.2–150 nmol/L	> 0.999 (*y* = 0.0318*x* + 0.0239)	0.35 nmol/L/–	[110]
ESI–MS (LC–MS/MS)	Rat brain microdialysate	0.05–5.00 nmol/L	0.999	0.05 fmol/0.25 fmol	[111]
MS (LC–MS/MS)	Murine brain	68.4–6840 pmol/L	0.9999 (*y* = 0.0043*x* + 0.0054)	1.37 pmol/L/6.85 pmol/L	[112]
MS (UPLC–MS/MS)	Rat cerebrospinal fluid	0.17–34.20 pmol/L	0.999	–/1.70 pmol/L	[113]
MS (LC–ESI–MS/MS)	Rat brain microdialysate	0.1–50 nmol/L	0.9994 (*x* = 177.49*x* + 53.79)	0.07 nmol/L/0.1 nmol/L	[114]
MS (LC–ESI–MS/MS)	Cerebral mice microdialysis	0.05–10 nmol/L	> 0.996	0.02 nmol/L/0.05 nmol/L	[115]
FLD	Human serum	0.01–80 µmol/L 80–200 µmol/L	0.9961 0.991	2.7 nmol/L/–	[116]
FLD	Synthetic	0.5–60 μmol/L	0.9976	0.5 μmol/L/–	[117]
MS (UPLC–MS/MS)	Human peripheral blood mononuclear cells	3.42–34.2 pmol/L	0.9993 (*y* = 2.2521*x* + 0.0038)	0.034 pmol/–	[118]
MS (UHPLC–MS)	Plant parts of different Atriplex species	3.42–136.8 nmol/L	0.99 (*y* = 1.26 × 10^−7^*x* − 4.75 × 10^–6^)	1.37 nmol/L/3.42 nmol/L	[119]
FLD	Synthetic	1–10 µmol/L	–	0.317 µmol/L/1.05 µmol/L	[120]
SERS	Secretion of ACh from PC12 cells	0.0001–100 μmol/L	> 0.9885	10 fmol/L/–	[121]
FLD	Human plasma	0.01–100 μmol/L	0.9946	8.9 nmol/L/–	[122]
MS (FT–ICR–MS)	Lungs of asthma model mice	1.56 to 0.20 pmol 100 to 0.20 pmol	0.997 0.9865	0.20 pmol/–	[123]
MS (LC–MS/MS)	Murine microdialysate	0.34–683.96 pmol/L	0.9988	0.17 pmol/L/0.34 pmol/L	[124]
MS (IC-MS/MS)	Feed, blood and urine of animals	3.42–683.96 pmol/L	0.9993 (*y* = 92464*x* + 28,182)	–/6.84 pmol/L	[125]
MS (LC–MS/MS)	Human cerebrospinal fluid	34.20–1368 pmol/L	0.9995 (*y* = 0.00020*x* + 0.00049)	–/34.2 pmol/L	[126]
MS (UHPLC-ESI–MS/MS)	Rat brain	0.171–17.1 nmol/L	0.9998 (*y* = 0.0548*x* − 0.0001)	68.4 pmol/L/171 pmol/L	[127]

*GC* gas chromatography, *MS* mass spectrometry, *LC* liquid chromatography, *SP* spectrophotometry, *HPLC* high performance liquid chromatography, *APCI* atmospheric pressure chemical ionization, *ESI* electrospray ionization, *HILIC* hydrophilic interaction liquid chromatography, *NIR-FLD* near infrared fluorescence detector, *MALDI* matrix-assisted laser desorption/ionization, *TOF* time of flight, *UPLC* ultra-performance liquid chromatography, *FLD* fluorescence detector, *UHPLC* ultra-high-performance liquid chromatography, *SERS* surface-enhanced Raman spectroscopy, *FT-ICR* Fourier-transform ion cyclotron resonance, *IC* ion chromatography

Another spectroscopic method—NMR—was applied for studies concerning ACh. It is mostly used for the standard analysis of compound structural conformation [56–60]. Communications of the NMR studies of hydrogen bonding of ACh [61], ACh in the form of halides [62, 63] or ACh in the presence of anesthetics [64] are also presented in the available literature. Moreover, the NMR spectroscopy was used to examine enzymatic hydrolysis of ACh [65], to assess ACh status in synaptic vesicles [66], to evaluate the purity of ACh extracts from oat seedings [44], to study the solvent effect on ACh [67], to study intramolecular dynamics of polycrystalline acetylcholine chloride [68], or to control some kinetic phenomena [69]. According to the researchers, successful determination of ACh can be performed with RS [55, 55, 70–72], IR [70, 71, 73–75], FS [76–79], and SP [80]. Application of several microscopic techniques such as electron tunneling spectroscopy [49], magnetic resonance spectroscopy (MRS) [81], magnetic resonance spectroscopy (MRI) [82], video-enhanced contrast microscopy (VECM) [83], atomic force microscopy (AFM) [84–86], miniature end-plate potentials (MEPPs) [87], radiochemical technique (RT), and real-time video-microscopy (TRVM) [88] for ACh determination and imaging is as well discussed in the literature.

## Sensors Technologies and Flow Techniques

**Fig. 5:**
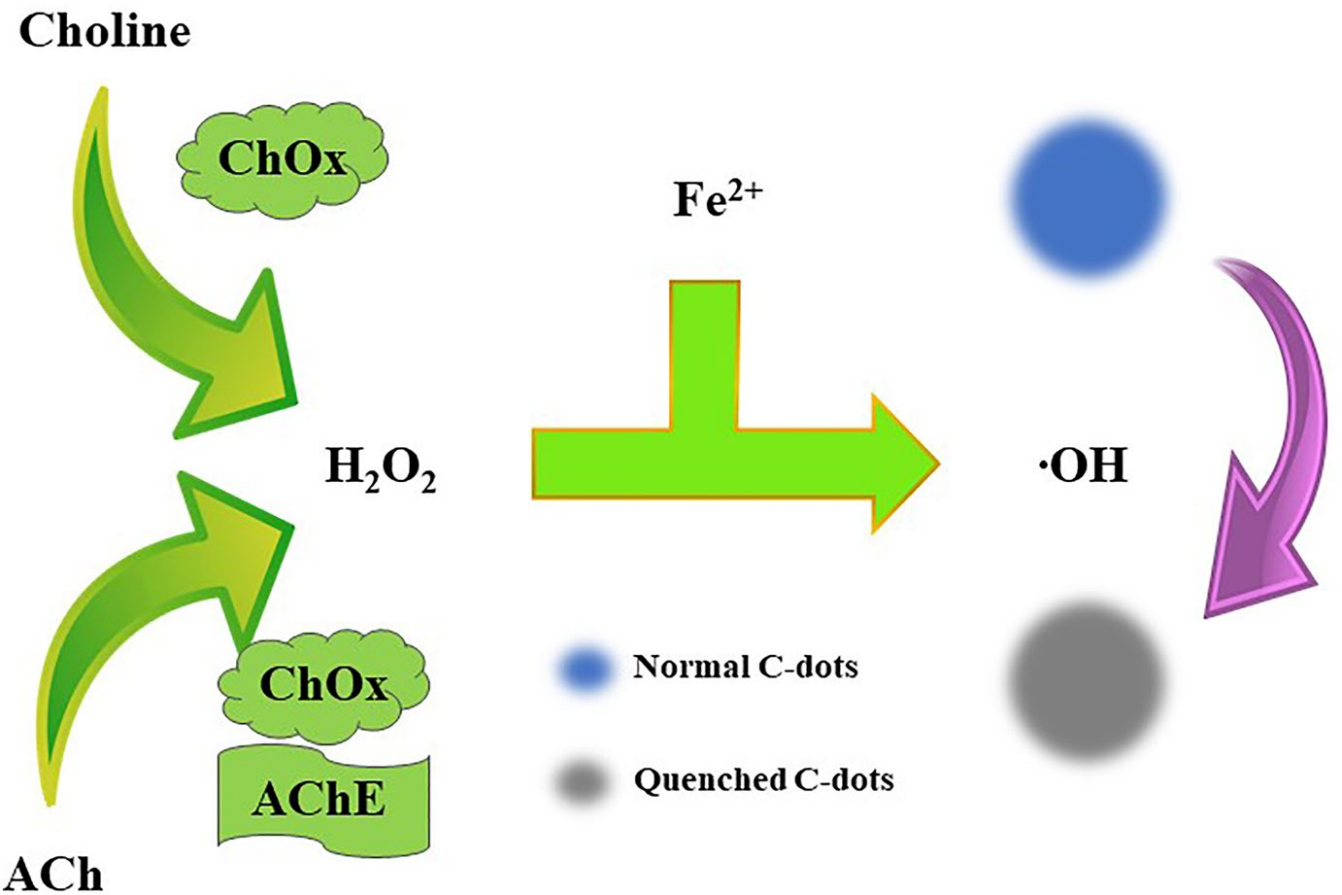
Scheme of sensor used for the detection of Ch and ACh using C-dots. Based on [117], where: *ChOx* choline oxidase and *AChE* acetylcholinesterase

**Fig. 6:**
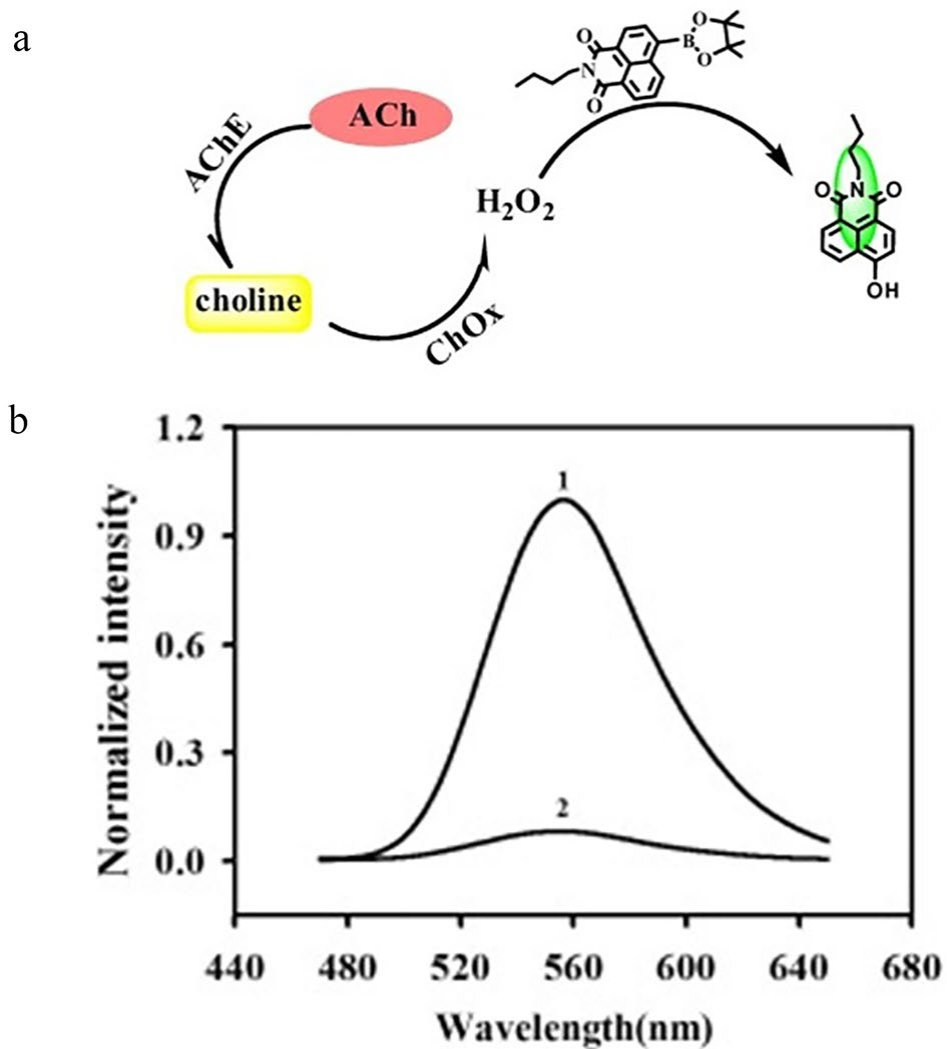
**a** Illustration showing the sensing probe to detect ACh. Reprinted from Analytical Biochemistry, 465, Chang Liu, Youming Shen, Peng Yin, Lidong Li, Meiling Liu, Youyu Zhang, Haitao Li, Shouzhuo Yao, Sensitive detection of acetylcholine based on a novel boronate intramolecular charge transfer fluorescence probe, 172–178, Copyright (2014), with permission from Elsevier. **b** Exemplary normalized FS spectra of the probe. Reprinted from Analytical Biochemistry, 465, Chang Liu, Youming Shen, Peng Yin, Lidong Li, Meiling Liu, Youyu Zhang, Haitao Li, Shouzhuo Yao, Sensitive detection of acetylcholine based on a novel boronate intramolecular charge transfer fluorescence probe, 172–178, Copyright (2014), with permission from Elsevier, where: *ChOx* choline oxidase and *AChE* acetylcholinesterase.

**Fig. 7:**
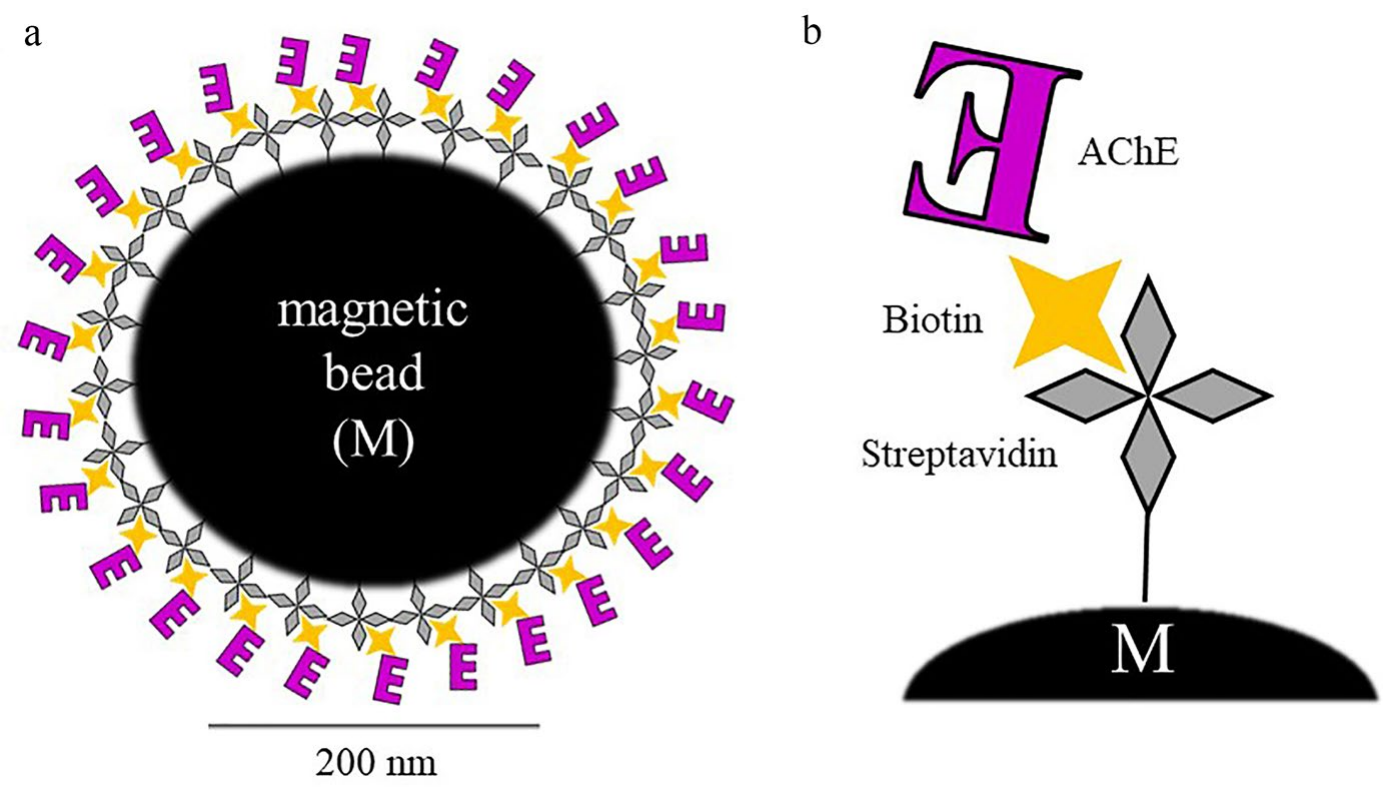
**a** Schematic illustration of formation process of a complex of magnetic nano-bead (M) with AChE. **b** The streptavidin-coated M was conjugated with biotinylated AChE. The complex has been magnetically immobilized in every ion image detection area. Based on [88]

**Fig. 8:**
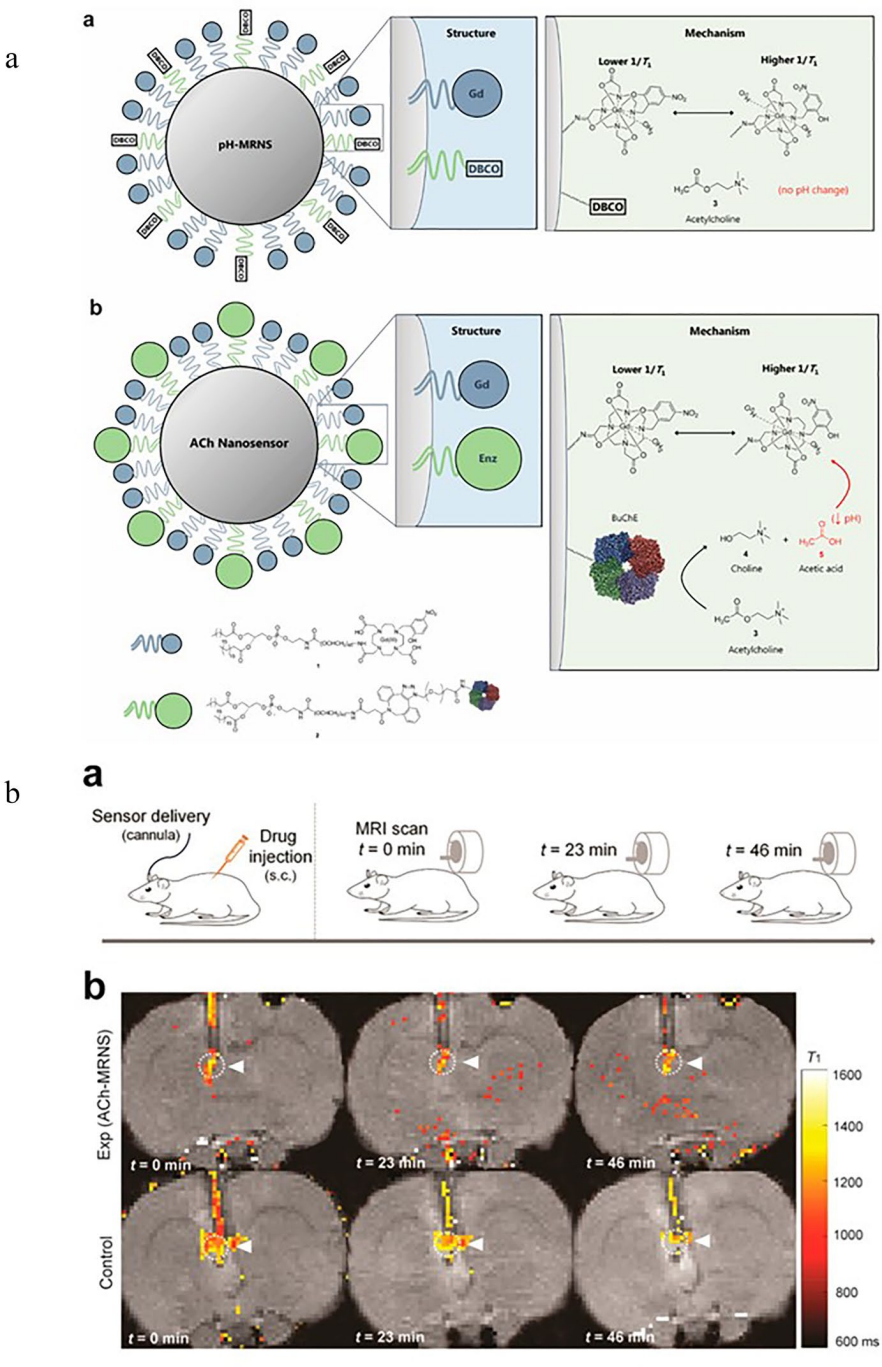
**a** Schematic illustration of the structure and mechanism of constructed nanosensor. **a** pH-MRNS. pH-sensitive contrast agents were conjugated to the DSPE-PEG [1, 2-distearoyl-sn-glycero-3-phosphoethanolamine-Poly(ethylene glycol)] lipids and coated on the surface of the lipophilic core [ACh is not hydrolyzed to alter local pH without coimmobilized BuChE (butyrylcholinesterase)]. **b** ACh-MRNS. pH-sensitive contrast agents and BuChE were covalently conjugated to the DSPE-PEG lipids and coated on the surface of the lipophilic core (the BuChE catalyzes the hydrolysis of ACh to Ch and acetic acid, resulting in a drop in local pH, which triggers a conformational switch of the contrast agent—one more water molecule coordinated to one Gd(III) chelate in acidic conditions, which leads to an increased contrast agent relaxation rate). Reprinted with permission from Ref. [129]. Copyright (2018) American Chemical Society. **b** ACh detection in vivo. a—Experimental procedure—subcutaneous administration of drug and nanosensor delivery through the cannula and three MR scans at different times. b—Exemplary coronal brain slices presenting ACh detection at different times. Reprinted with permission from Ref. [129]. Copyright (2018) American Chemical Society

There is some research focusing on the sensor detection of ACh with spectroscopic techniques. The most popular detection technique, due to its native sensitivity, is FS. Sensors designed for this approach are based on the host–guest complexes of ACh and the hosting molecules [77, 103, 108]. Another approach is the application of particles whose fluorescence properties are sensitive to the products of ACh enzymatic reaction induced by AChE. For such studies, carbon-based probes like carbon-dots (C-dots) [117] (see Fig. 5, [117]) or graphene oxide-nanoconjugates [78] are used, as well as gold nanoclusters (AuNCs) [120] and other nonspecific fluorescent molecules [79, 116, 122]. Figure 6 shows the developed sensor together with an example of a normalized FS spectrum [116]. ACh sensing with the FS facilitates detection in ranges of µM to nM level. SERS is another spectroscopic technique applied for the discussed purpose. The authors report ACh detection levels of 10 fM [121] and 1 aM [55] when SERS is used. For the sensing of ACh, an ion image sensor was also designed [128]. Biotinylated AChE and streptavidin-conjugated magnetic nano-bead were components of the ACh-sensitive layer, see Fig. 7 [128]. In 2018, nanoparticle sensors serving as MRI contrast agents for ACh detection were also reported [129] (Fig. 8 shows the diagram of the sensor operation and exemplary results).

Flow analysis is characterized by several features supporting its widespread use. The following advantages should be mentioned: simple construction of the system, maintained constant measurement conditions, reduced consumption of samples and reagents, the possibility of connecting the system with another measuring instrument, computerization of the system, high repeatability of recorded signals, and obtaining many signals during single analysis. The use of flow techniques for the determination of ACh using spectroscopic techniques has not been popular so far. Only three articles in this area have been published [63, 80, 121].

**Fig. 9:**
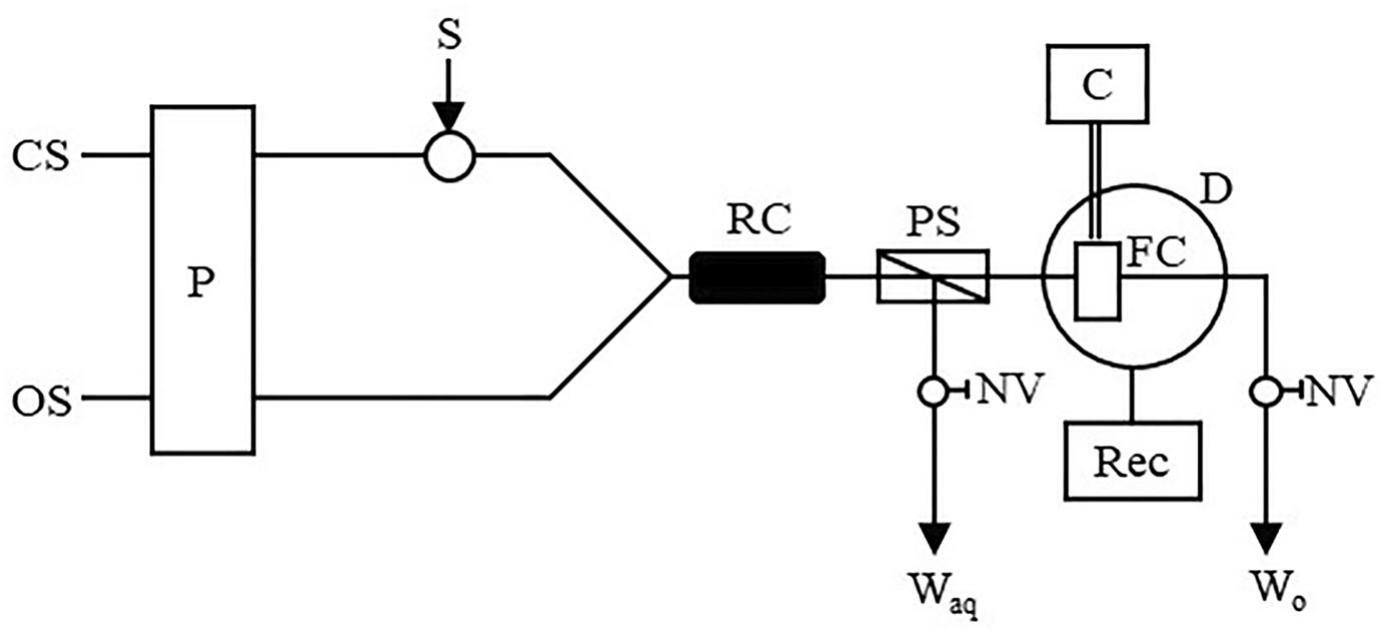
Diagram of the flow system used to determination of ACh, where: *CS* carrier solution (buffer at pH 11), *OS* extractant (TBPE H solution in dichloroethane), *P* pump (flow rate equal to 0.8 mL/min), *S* sample injection (140 µL), *RC* reaction coil (3 m × 0.5 mm ID), *PS* phase separator, *FC* flow cell (8 µL), *C* circulator, *D* detector, *Rec* recorder, *NV* needle valve, *W*_*aq*_ aqueous waste, *W*_*o*_ organic waste (based on [80, 93])

In 1991, Sakai et al. presented two reports on the spectrophotometric determination of ACh and Ch using the flow-injection technique [80, 93]. This method involved the use of a reaction with tetrabromophenolphthalein ethyl ester (TBPE H). It was based on the creation of ion association compounds with TBPE H and thermochromism of ion associates in the organic phase. In more detail, ACh and Ch, due to possessing a quaternary ammonium structure, are able to react with an association reagent and create a blue ion association complex. The method developed in this way allowed for the improvement of the selectivity. A schematic diagram of the built two-line flow-injection system is presented in the Fig. 9.

The sample solution was injected into the buffered carrier solution (pH 11) and then mixed with an extractant (TBPE H solution in dichloroethane), used for ion-pair extraction. The organic phase was separated using a porous polytetrafluoroethylene (PTFE) membrane [phase separator (PS)]. This phase was then directed through a microfluidic chamber (temperature controlled at 45 °C), where the absorbance was measured. The blue ion ACh/Ch association complexes did not change the absorbance value with temperature changes. On the other hand, the absorbance of red amine complexes disappears at a temperature kept at 45 °C. The developed method was characterized by acceptable validation parameters.

**Fig. 10:**
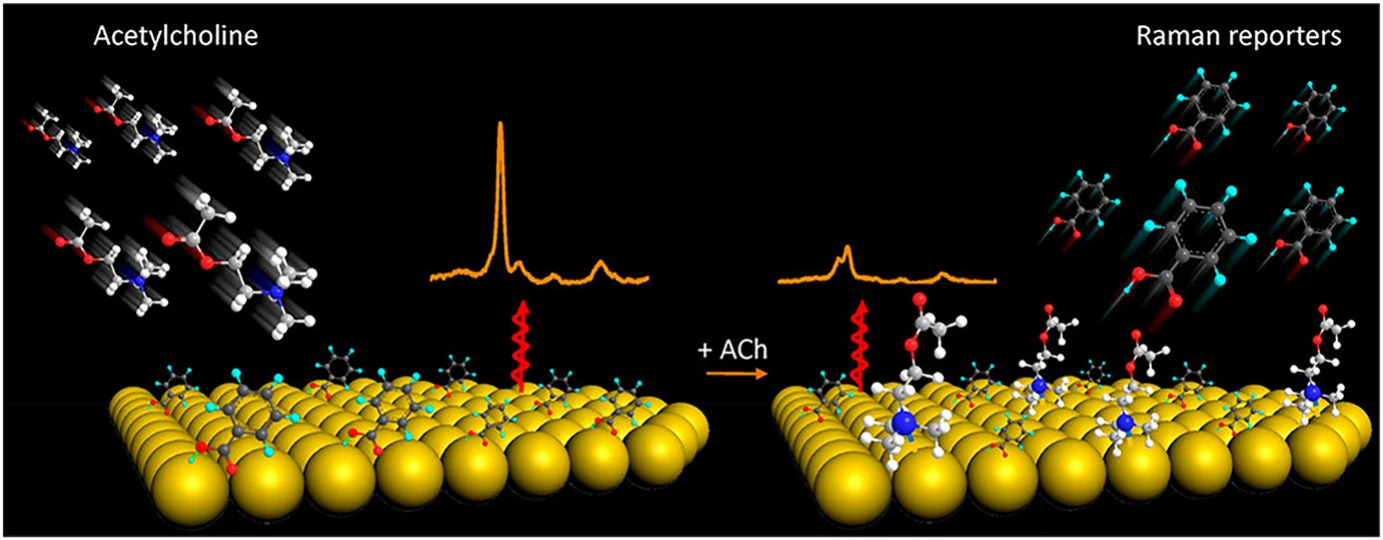
Schematic diagram presenting quaternary-ammonium-modulated surface-enhanced Raman spectroscopy (QAM-SERS) effect applied in a constructed microfluidic device. Reprinted with permission from Ref. [121]. Copyright (2020) American Chemical Society

One of the newer solutions published in recent years is based on using a microfluidic technique coupled with SERS spectroscopy to determine ACh [121]. In this work, a completely new type of effect was proposed—the quaternary-ammonium-modulated surface-enhanced Raman spectroscopy (QAM-SERS) effect. The QA compounds (like ACh) cause a concentration-dependent modulation of the SERS signal intensity of the Raman reporter (Fig. 10). In this effect, the ionic bonds between QA nitrogen atoms and nanoparticles (NPs), such as Ag or Au, are created.

**Fig. 11:**
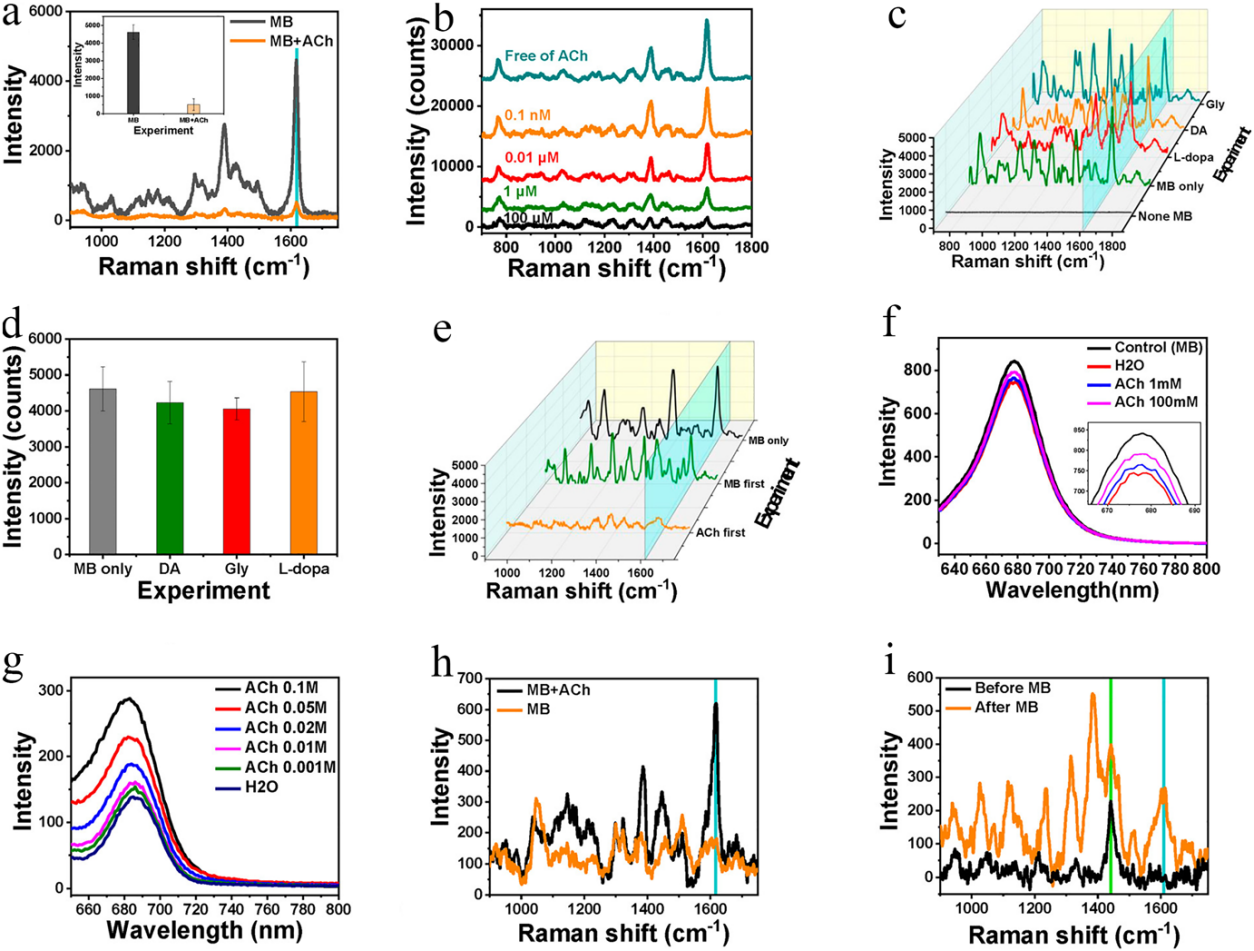
**a** SERS spectra of methylene blue (MB), with and without ACh (the inset shows the SERS intensity at the 1617 cm^–1^ peak), **b** SERS spectra of MB (10 μM) after addition to a different concentration of ACh, **c** SERS spectra of MB after addition of L-dopa, dopamine (DA), and glycine (Gly), **d** SERS intensities at 1617 cm^–1^ peak in **c**, **e**. SERS spectra of MB when MB and ACh were added to the SERS-active substrate in different sequences. **f**–**i** Fluorescence and SERS spectra for testing the interaction between ACh and MB on the Ag NPs: **f** FS spectra of MB molecules remained in the water, **g** FS spectra of MB in ACh solution, **h** SERS determination of MB in the outflow, and **i** SERS intensity of ACh with and without the MB solution. The concentration of MB was 1 μM, and the concentrations of ACh, DA, Gly, and L-dopa were 1 mM (unless expressly noted). Reprinted with permission from Ref. [121]. Copyright (2020) American Chemical Society

In the first step, the SERS-active substrate is prepared by electrostatic self-assembly of Ag NPs on the bottom of the microfluidics system. Chips were prepared with PDMS (polydimethylsiloxane) material, while microfluidic channels with a width of 500 μm and height of 100 μm were made on the surface. Then, glass slides were attached to the surface of the chips. The mixture consisting of the ACh solution and the Raman reporter is placed on the SERS substrate with which it interacts. The analytical signal from the Raman reporter decreases in direct proportion to the increase in analyte concentrations. Based on this effect, an improvement in the LOD (10 fM) and high sensitivity was obtained, as well as an ultrawide dynamic range (10 orders of magnitude) was observed. Example SERS spectra and other results are shown in Fig. 11. The time-dependent secretion of ACh from living PC12 cells was carried out to test the method in the context of in vitro ACh dynamic changes. In the future, the discovered effect may turn out to be a universal strategy for the determination of ACh in biological samples, or it may prove to be an inspiration to develop research in this direction, especially with the use of the microfluidic technique.

## Single/Multi Component Analysis

Works published so far in the field of spectroscopic determination of ACh include both single analysis and multi component analysis. Spectroscopic techniques for single (ACh) determination and their analytical characteristics have already been presented in Table 1.

However, it should be noticed that a lot of the developed spectroscopic methods allow for the simultaneous determination of ACh and one or more different analytes. Table 2 summarizes the most important information about multi component analyses including spectroscopic approaches. It can be seen that the analyte most often determined together with ACh is its precursor: Ch. The most popular methods of simultaneous determination of ACh and Ch among spectroscopic techniques include MS coupled to chromatography techniques: GC [43, 46, 48, 90, 130–132], LC and HPLC [50, 91, 96, 99, 126, 133, 134], as well as matrix-assisted laser desorption/ionization time of flight (MALDI-TOF) MS [110]. Moreover, ACh and Ch are determined by applying FS [117, 120, 122], SP [94], or NMR [44]. GC–MS is a popular technique for determination of ACh and propionylcholine [135, 136], ACh, propionylcholine and butyrylcholine [137], and tellurium containing analogs of ACh and Ch [46]. Magnetic resonance spectroscopy MRS has been applied for ACh, phosphocholine, and betaine determination [81]. To carry out three and more component analyses, including the determination of ACh, Ch, and other analytes, such as betaine, dimethylglycine, butyrobetaine, 20-hydroxyecdysone, aspartic acid, asparagine, glutamic acid, glutamine, pyroglutamate, γ-aminobutyric acid, *N*-acetyl-l-aspartic acid, tryptophan, kynurenine, carnitine, and acetylcarnitine, chromatographic techniques coupled with MS are used. Most often it is GC–MS [45, 138] or LC–MS/MS [102, 124] techniques that are used, as well as UPLC–MS/MS [110], HILIC–MS/MS [104], UHPLC–MS [119], and UHPLC–ESI–MS/MS [127]. In addition to Ch, dopamine is an analyte often co-determined with ACh. In this field LC–MS [139], LC–MS/MS [100], LC–ESI–MS/MS [115], and SERS [55] are applied. It is worth noting that the determination of ACh and dopamine is also accompanied with serotonin, γ-aminobutyric acid, glutamate, adenosine, and/or 5-hydroxytryptamine. Multi component analyses also include simultaneous determination of ACh and neostigmine or ACh and histamine and its metabolites. Spectroscopic methods used for this purpose is a tandem MS coupled to LC [97] or UPLC [113], respectively.

**Table 2:** Spectroscopic techniques for multi component analyses, including determination of ACh and other analytes

Spectroscopic technique	Analyte(s) simultaneously determined with ACh	Sample	References
MS (GC–MS)	Ch	Synthetic	[48]
MS (GC–MS)	Ch	Mouse brain	[130]
MS (GC–MS)	Ch	Rat brain	[131]
MS (GC–MS)	Ch	Cat caudate tissue	[132]
MS (GC–MS)	Ch	Rat brain	[43]
MS (GC–MS)	Ch	Rat brain	[95]
MS (capillary GC–MS)	Ch	Canine brain and blood	[90]
MS (LC–MS)	Ch	Synthetic	[133]
MS (thermospray LC–MS)	Ch	Mouse brain	[91]
MS (LC–MS/MS)	Ch	Mice and rat brain microdialysates	[99]
MS (LC–MS/MS)	Ch	Human cerebrospinal fluid	[126]
MS (thermospray-HPLC–MS)	Ch	Mouse brain	[134]
MS (HPLC–MS)	Ch	Rat pheochromocytoma cell line (PC12)	[96]
MS (HPLC–ESI–MS)	Ch	Drugs	[50]
MS (MALDI-TOF MS)	Ch	Mouse brain cerebrospinal fluid	[109]
FS	Ch	Synthetic	[117]
FS	Ch	Synthetic	[120]
FS	Ch	Human plasma	[122]
NMR	Ch	Green oat	[44]
SP	Ch	Synthetic	[94]
MS (GC–MS)	Propionylcholine	Bull sperm	[135]
MS (GC–MS)	Propionylcholine	Human term placentae	[136]
MS (GC–MS)	Propionylcholine, butyrylcholine	Rat brain	[137]
MS (GC–MS)	Tellurium containing analogs of ACh and Ch	Mouse brain	[46]
MRS	Phosphocholine, betaine	Rat brain	[81]
MS (GC–MS)	Ch, arecoline	Mouse tissues	[138]
MS (GC–MS)	Ch, acroleine	Mouse brain	[45]
MS (LC–ESI/MS/MS)	Ch, (3-carboxylpropyl)-trimethylammonium	Rat brain microdialysates	[102]
MS (LC–MS/MS)	Ch, serotonin, 5-hydroxyindoleacetic acid, melatonin, dopamine, levodopa, 3-methoxytyramine, norepinephrine, epinephrine, γ-aminobutyric acid	Murine microdialysates	[124]
MS (UPLC–MS/MS)	Ch, betaine, dimethylglycine	Human plasma and urine	[110]
MS (HILIC–MS/MS)	Ch, butyrobetaine	Human liver	[104]
MS (UHPLC–MS)	Ch, betaine, 20-hydroxyecdysone	Plant parts of different Atriplex species	[119]
MS (UHPLC–ESI–MS/MS)	Ch, aspartic acid, asparagine, glutamic acid, glutamine, pyroglutamate, γ-aminobutyric acid, *N*-acetyl-l-aspartic acid, tryptophan, kynurenine	Rat brain	[127]
MS (IC–MS/MS)	Ch, carnitine, acetylcarnitine	Feed, blood, and urine of animals	[125]
MS (LC–MS)	Dopamine, serotonin, γ-aminobutyric acid, glutamate	Synthetic	[139]
MS (LC–MS/MS)	Dopamine D_3_ receptor antagonists	Rat brain microdialysates	[100]
MS (LC–ESI–MS/MS)	Dopamine, adenosine, 5-hydroxytryptamine	Cerebral mice microdialysates	[115]
SERS	Dopamine, serotonin, γ-aminobutyric acid, glutamate	Synthetic	[55]
MS (LC–MS/MS)	Neostigmine	Rat brain microdialysates	[97]
MS (UPLC–MS/MS)	Histamine and its metabolites	Rat cerebrospinal fluid	[113]

*GC* gas chromatography, *MS* mass spectrometry, *LC* liquid chromatography, *HPLC* high performance liquid chromatography, *MALDI* matrix-assisted laser desorption/ionization, *TOF* time of flight, *FS* fluorescence spectroscopy, *NMR* nuclear magnetic resonance, *SP* spectrophotometry, *UPLC* ultra-performance liquid chromatography, *HILIC* hydrophilic interaction liquid chromatography, *UHPLC* ultra-high-performance liquid chromatography, *IC* ion chromatography, *ESI* electrospray ionization, *SERS* surface-enhanced Raman spectroscopy

## Tested Samples and Extraction Procedures

**Fig. 12:**
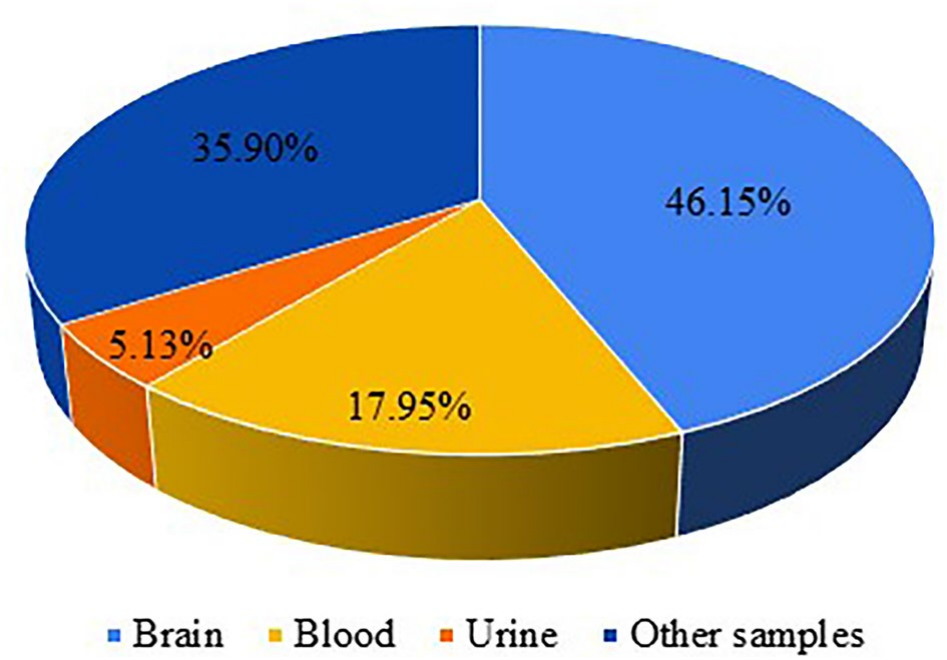
Types of real samples analyzed for ACh determination applying spectroscopic techniques. Other samples include liver, lungs, corneal epithelium, cerebrospinal fluid, PC12 cells, cell lysate and plant parts. Figure based on Table 1 data

Based on the works presented in the literature, it can be seen that the spectroscopic determination of ACh is carried out in various types of biological samples of human and animal origin (Table 1). Figure 12 shows that ACh is usually determined in brain samples. A similar trend is observed in multi component analyses (Table 2). The second most frequently analyzed sample type for ACh concentration is blood. Moreover, the indicated neurotransmitter is determined in urine samples and in other types of samples (liver, lungs, corneal epithelium, cerebrospinal fluid, PC12 cells, cell lysate, and plant parts).

**Fig. 13:**
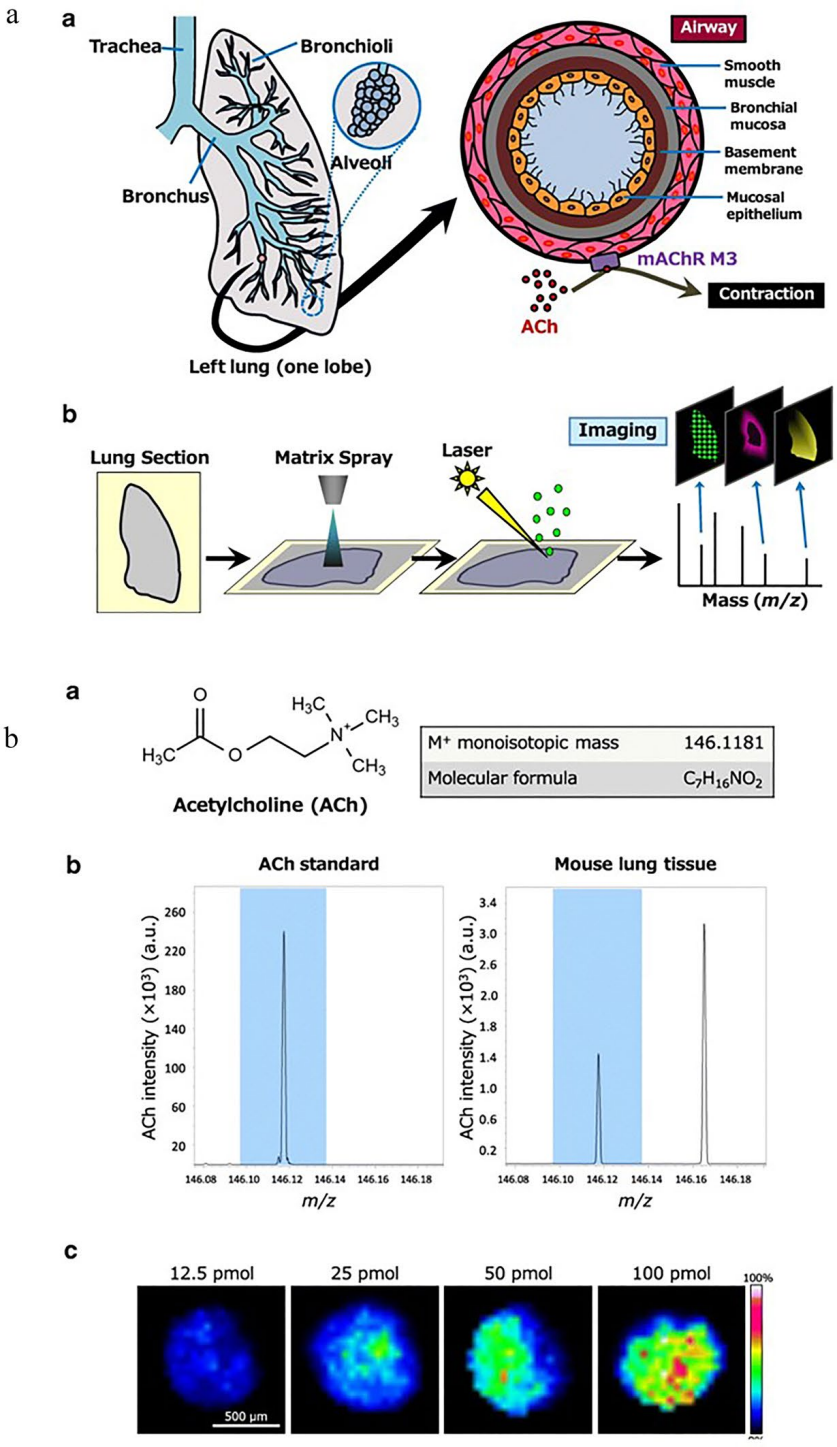
**a** Schematic illustration of the mouse lung anatomy and imaging MS. a—Mouse lung anatomy. b—Imaging MS (IMS), which allows visualization of the distribution and abundance of a target molecule in biological tissue. The figure is from an open access article distributed under the terms of the Creative Commons CC BY license. Copyright © 2020. Springer, Nature. **b** Signal of ACh and amount-dependent signal intensity. a—ACh chemical structure (the theoretical exact mass is 146.1181). b—Comparison of ACh signal peaks for the standard and lung tissue. c—The ACh abundance dilution of the standard on lung tissue in spots visualized by Fourier transform ion cyclotron resonance mass spectrometry (FT-ICR-MS). The figure is from an open access article distributed under the terms of the Creative Commons CC BY license. Copyright © 2020. Springer, Nature

Preparation of samples for ACh determination using spectroscopic methods very often requires extraction process [42–46, 69, 73, 80, 89–91, 93, 95, 96, 104, 106, 110, 112, 119, 119, 125–127, 131, 132, 135–138, 140–143]. Among the extraction methods used, simple ion pair extraction [80, 131] and multistep liquid extraction [45, 46, 73, 95, 104, 106] should be mentioned. In the works discussed in this review article, sample preparation is often accompanied by a microdialysis concept. This process, based on separation of small and large molecules by diffusion through a selectively permeable membrane, was applied, for example, for nuclear magnetic resonance studies of the enzymatic hydrolysis of ACh in monkey brains [65], studies of drug effects on the release of endogenous ACh in vivo [92], quantitative analysis of ACh in rat brain [144], or simultaneous determination of ACh and Ch in mouse brain CSF [109]. Samples that were analyzed as dialysates are indicated in Tables 1 and 2. In several spectroscopic methods for the determination of ACh described in the literature, no special sample preparation is needed: sometimes only dilution with water [50, 116] or buffer [98] is required. Moreover, in some cases, slide-mounted sections of biological samples are analyzed directly after matrix spraying [123]. This approach was applied in imaging MS to visualize increased ACh in lungs of asthma model mice (Fig. 13).

## Conclusions and Future Perspectives

The use of spectroscopic techniques to detect and determine ACh, especially in biological samples from animal models, turned out to be an alternative to commonly used chromatographic methods, particularly HPLC-ED, and other methods like ELISA, radiometric assays, or potentiometric methods. The most frequently used technique was MS, allowing reliable determination of results for ACh after prior chromatographic separation. This application was characterized by high sensitivity, low limits of detection and quantification, as well as a wide dynamic range. The main disadvantage when coupling the mass spectrometer with the chromatographic system is the need for the often complicated preparation of biological samples to remove the complex sample matrix. The second most commonly used technique was NMR, but it was primarily used for structural studies and to determine the connections of ACh with the appropriate receptors.

When FS was applied, it showed very good applicability, mainly in properly developed sensors and probes. In the case of using the SERS technique, very low detection limits at the aM level were demonstrated due to the ability to detect even single molecules. In this area, it is necessary to use SERS-active substrates to amplify the Raman analytical signal.

ACh is determined as a single analyte, together with Ch, or in the presence of other neurotransmitters in the case of multiplexed analysis using separation techniques. The vast majority of investigations focus on studying dialysates, brain tissues from laboratory animals, and samples of body fluids, such as blood, serum, or urine. Until now, only a few reports focused on the use of flow techniques with constructed, dedicated devices and microfluidics systems.

The use of the discovered QAM-SERS effect may prove to be highly prospective. It can be the basis for the creation of new biosensors in combination with the microfluidic technique or lab-on-a-chip systems. The technique supporting the SERS effect seems to be FS, which gives additional valuable information from the analytical point of view. Therefore, both spectroscopic methods can be integrated into a single system. Development in multiplex analysis is also forecasted, enabling the determination of several other neurotransmitters, in addition to ACh, in a single analytical procedure. The use of additional modules in appropriate microfluidic systems, allowing for proper sample preparation with the possibility of their separation directly on the chip, through the placement or generation in the flow microcolumns, may prove effective in this area. Based on the scientific literature, it is also predicted to integrate such chips with mass spectrometry. In the case of obtaining complex analytical signals from various spectroscopic techniques, it may be helpful to adapt already existing chemometric methods and algorithms.

Studies presented in this review paper and future innovations will allow for fast and reliable determination of ACh and its monitoring in tissue or cell cultures. In this way, approaches based on spectroscopic techniques can be a better alternative to the currently used separation methods, such as liquid chromatography, which are perfect for basic research due to lower costs of analyses and the apparatus itself.

## Data Availability

The data supporting this study’s findings are openly available on the internet, as in the reference stated below. The authors also confirm that the data and materials supporting the findings of this study are available within the article.

## References

[1] Niyonambaza SD, Kumar P, Xing P (2019). A review of neurotransmitters sensing methods for neuro-engineering research. Appl Sci.

[2] Tracey DJ, Paxinos G, Stone J (1995). Neurotransmitters in the human brain.

[3] Duncan JS (2002). Neurotransmitters, drugs and brain function. Br J Clin Pharmacol.

[4] Herlenius E, Lagercrantz H, Peebles DM, Lagercrantz H, Ment LR, Hanson MA (2010). Neurotransmitters and neuromodulators. The newborn brain: neuroscience and clinical applications.

[5] Wu Z, Lin D, Li Y (2022). Pushing the frontiers: tools for monitoring neurotransmitters and neuromodulators. Nat Rev Neurosci.

[6] Südhof TC, Starke K (2008). Pharmacology of neurotransmitter release.

[7] Colangelo C, Shichkova P, Keller D (2019). Cellular, synaptic and network effects of acetylcholine in the neocortex. Front Neural Circ.

[8] Rand JB (2007) Acetylcholine. WormBook 1–21. 10.1895/wormbook.1.131.1 10.1895/wormbook.1.131.1 PMC4781110 18050502

[9] Brown DA (2019). Acetylcholine and cholinergic receptors. Brain Neurosci Adv.

[10] Pepeu G, Giovannini MG (2004). Changes in acetylcholine extracellular levels during cognitive processes. Learn Mem.

[11] Twyman RM, Squire LR (2009). Neutrotransmission and neuromodulation: acetylcholine. Encyclopedia of neuroscience.

[12] (2022) Acetylcholine|Definition, Function, & Facts|Britannica. https://www.britannica.com/science/acetylcholine. Accessed 26 Jan 2023

[13] Schliebs R, Arendt T (2011). The cholinergic system in aging and neuronal degeneration. Behav Brain Res.

[14] Bertrand D, Wallace TL (2020). A review of the cholinergic system and therapeutic approaches to treat brain disorders. Curr Top Behav Neurosci.

[15] Tiwari P, Dwivedi S, Singh MP (2013). Basic and modern concepts on cholinergic receptor: a review. Asian Pac J Trop Dis.

[16] Picciotto MR, Jentsch JD, Alreja M (2002). Acetylcholine. Neuropsychopharmacology—5th generation of progress.

[17] Van der Zee EA, Keijser JN (2011). Localization of pre- and postsynaptic cholinergic markers in rodent forebrain: a brief history and comparison of rat and mouse. Behav Brain Res.

[18] Picciotto MR, Higley MJ, Mineur YS (2012). Acetylcholine as a neuromodulator: cholinergic signaling shapes nervous system function and behavior. Neuron.

[19] Woolf NJ, Butcher LL (2011). Cholinergic systems mediate action from movement to higher consciousness. Behav Brain Res.

[20] Takács VT, Cserép C, Schlingloff D (2018). Co-transmission of acetylcholine and GABA regulates hippocampal states. Nat Commun.

[21] Sam C, Bordoni B (2023). Physiology, acetylcholine.

[22] De Bundel D, Sarre S, Van Eeckhaut A (2008). Critical evaluation of acetylcholine determination in rat brain microdialysates using ion-pair liquid chromatography with amperometric detection. Sensors (Basel).

[23] Ahmed NY, Knowles R, Dehorter N (2019). New insights into cholinergic neuron diversity. Front Mol Neurosci.

[24] Klinkenberg I, Sambeth A, Blokland A (2011). Acetylcholine and attention. Behav Brain Res.

[25] Phillips PA, Yang L, Shulkes A (2010). Pancreatic stellate cells produce acetylcholine and may play a role in pancreatic exocrine secretion. Proc Natl Acad Sci U S A.

[26] Bazzu G, Biosa A, Farina D (2012). Brain microdialysis in freely moving animals. Methods Mol Biol.

[27] Anderzhanova E, Wotjak CT (2013). Brain microdialysis and its applications in experimental neurochemistry. Cell Tissue Res.

[28] Song P, Hershey ND, Mabrouk OS (2012). Mass spectrometry “sensor” for in vivo acetylcholine monitoring. Anal Chem.

[29] Świt P, Herian M, Gołembiowska K (2021). Improvement of analytical results quality in neuroscience—good methodology practice in the acetylcholine determination. Microchem J.

[30] Potter PE, Meek JL, Neff NH (1983). Acetylcholine and choline in neuronal tissue measured by HPLC with electrochemical detection. J Neurochem.

[31] Asano M, Miyauchil T, Kato T (1986). Determination of acetylcholine and choline in rat brain tissue by liquid chromatography/electrochemistry using an immobilized enzyme post column reactor. J Liq Chromatogr.

[32] Stadler H, Nesselhut T (1986). Simple and rapid measurement of acetylcholine and choline by HPLC and enzymatic-electrochemical detection. Neurochem Int.

[33] Beley A, Zekhnini A, Lartillot S (1987). Improved method for determination of acetylcholine, choline, and other biogenic amines in a single brain tissue sample using high performance liquid chromatography and electrochemical detection. J Liq Chromatogr.

[34] Damsma G, van Bueren DL, Westerink BHC, Horn AS (1987). Determination of acetylcholine and choline in the femtomole range by means of HPLC, a post-column enzyme reactor, and electrochemical detection. Chromatographia.

[35] Murai S, Miyate H, Saito H (1989). Simple determination of acetylcholine and choline within 4 min by HPLC-ECD and immobilized enzyme column in mice brain areas. J Pharmacol Methods.

[36] Fujiki Y, Ikeda Y, Okuyama S (1990). Determination of acetylcholine and choline in human plasma using high-performance liquid chromatography combined with an immobilized enzyme reactor. J Liq Chromatogr.

[37] Guerrieri A, Palmisano F (2001). An acetylcholinesterase/choline oxidase-based amperometric biosensors as a liquid chromatography detector for acetylcholine and choline determination in brain tissue homogenates. Anal Chem.

[38] Sotoyama H, Zhu Y, Gitzen JF, et al (2002) Feasibility of ion-pair reversed-phase liquid chromatography/electrochemistry detection for determination of acetylcholine in microdialysates collected without acetylcholinesterase inhibitors. Curr Sep

[39] Zackheim JA, Abercrombie ED, Wang JQ (2003). HPLC/EC detection and quantification of acetylcholine in dialysates. Drugs of abuse: neurological reviews and protocols.

[40] Aono Y, Watanabe Y, Ishikawa M (2019). In vivo neurochemical evidence that stimulation of accumbal GABAA and GABAB receptors each reduce acetylcholine efflux without affecting dopamine efflux in the nucleus accumbens of freely moving rats. Synapse.

[41] Nirogi R, Mudigonda K, Kandikere V, Ponnamaneni R (2010). Quantification of acetylcholine, an essential neurotransmitter, in brain microdialysis samples by liquid chromatography mass spectrometry. Biomed Chromatogr.

[42] Stanaszek PM, Snell JF, O’Neill JJ (1977). Isolation, extraction, and measurement of acetylcholine from *Lactobacillus plantarum*. Appl Environ Microbiol.

[43] Khandelwal JK, Szilagyi PI, Barker LA, Green JP (1981). Simultaneous measurement of acetylcholine and choline in brain by pyrolysis-gas chromatography–mass spectrometry. Eur J Pharmacol.

[44] Tretyn A, Bobkiewicz W, Tretyn M, Michalski L (1987). The identification of acetylcholine and choline in oat seedlings by gas chromatography and nuclear magnetic resonance (NMR). Acta Soc Bot Pol.

[45] Patterson TA, Kosh JW (1992). Simultaneous quantitation of arecoline, acetylcholine, and choline in tissue using gas chromatography/electron impact mass spectrometry. Biol Mass Spectrom.

[46] Harris SE, Silks LA, Dunlap RB (1993). Synthesis of novel tellurium containing analogues of choline and acetylcholine and their quantitation by pyrolysis-gas chromatography-mass spectrometry. J Chromatogr A.

[47] Zhu Y, Wong PS, Cregor M (2000). In vivo microdialysis and reverse phase ion pair liquid chromatography/tandem mass spectrometry for the determination and identification of acetylcholine and related compounds in rat brain. Rapid Commun Mass Spectrom.

[48] Jenden DJ, Roch M, Booth RA (1973). Simultaneous measurement of endogenous and deuterium-labeled tracer variants of choline and acetylcholine in subpicomole quantities by gas chromatography/mass spectrometry. Anal Biochem.

[49] Aslanian D, de Cheveigné S (1982). Inelastic electron tunneling spectroscopic study of interaction of acetylcholine and beta-methyl acetylcholine with alumina surface. Mol Pharmacol.

[50] Dunphy R, Burinsky DJ (2003). Detection of choline and acetylcholine in a pharmaceutical preparation using high-performance liquid chromatography/electrospray ionization mass spectrometry. J Pharm Biomed Anal.

[51] Ohashi M, Lino T, Takahashi T (1990). Cluster ions in the secondary ion mass spectrometry of choline and acetylcholine halides. Org Mass Spectrom.

[52] Ikarashi Y, Itoh K, Matsuura K, Maruyama Y (1990). Quantitative determination of acetylcholine in rat brain regions by liquid chromatography/mass spectrometry with FRIT-FAB interface. Jpn J Clin Chem.

[53] Shackman HM, Shou M, Cellar NA (2007). Microdialysis coupled on-line to capillary liquid chromatography with tandem mass spectrometry for monitoring acetylcholine in vivo. J Neurosci Methods.

[54] Sugiura Y, Zaima N, Setou M (2012). Visualization of acetylcholine distribution in central nervous system tissue sections by tandem imaging mass spectrometry. Anal Bioanal Chem.

[55] Lee W, Kang B-H, Yang H (2021). Spread spectrum SERS allows label-free detection of attomolar neurotransmitters. Nat Commun.

[56] Cushley RJ, Mautner HG (1970). NMR studies on the conformation of acetylcholine isologues. Tetrahedron.

[57] Partington P, Feeney J, Burgen AS (1972). The conformation of acetylcholine and related compounds in aqueous solution as studied by nuclear magnetic resonance spectroscopy. Mol Pharmacol.

[58] Jones GP, Roberts RT, Anderton KJ, Ahmed AMI (1972). Nuclear magnetic resonance diffusion and relaxation time study of acetylcholine. J Chem Soc Faraday Trans.

[59] Chynoweth KR, Ternai B, Simeral LS, Maciel GE (1973). Nuclear magnetic resonance studies of the conformation and electron distributions in nicotine and in acetylcholine. Mol Pharmacol.

[60] Cassidei L, Sciacovelli O (1981). Conformational analysis of the C(6)-O(1)-C(5)-C(4) fragment in acetylcholine by carbon-13 NMR spectroscopy. J Am Chem Soc.

[61] Harmon KM, Bulgarella JA (1995). Hydrogen bonding. Part 61. FT-NMR study of acetylcholine and tetrapropylammonium ion; conformation and stoichiometry of hydration of tetrapropylammonium ion in aqueous solution. J Mol Struct.

[62] Harmon KM, Avci GF, Desantis NJ, Thiel AC (1985). Hydrogen bonding: Part 19. IR and NMR study of the lower hydrates of choline fluoride and acetylcholine chloride. J Mol Struct.

[63] Harmon KM, Akin AC, Avci GF (1991). Hydrogen bonding: Part 33. NMR study of the hydration of choline and acetylcholine halides. J Mol Struct.

[64] Akin AC, Harmon KM (1994). Hydrogen bonding Part 54. NMR study of the effects of anesthetics on hydration of choline, acetylcholine and tetraethylammonium halides in aqueous solution. J Mol Struct.

[65] Krishnan KS, Balaram P (1977). Nuclear magnetic resonance studies of the enzymatic hydrolysis of acetylcholine: a critical comment. Mol Pharmacol.

[66] Stadler H, Füldner HH (1980). Proton NMR detection of acetylcholine status in synaptic vesicles. Nature.

[67] Sega EM, Tormena CF, de Oliveira PR (2006). Solvent effects in the 2JHH, 3JHH, 1JNC and 2JNC coupling constants in the NMR spectrum of acetylcholine chloride. J Mol Struct.

[68] Świergiel J, Piślewski N, Medycki W (2004). 1H NMR study of molecular dynamics of acetylcholine chloride. Appl Magn Reson.

[69] Hall H, Cuellar-Baena S, Denisov V, Kirik D (2013). Development of NMR spectroscopic methods for dynamic detection of acetylcholine synthesis by choline acetyltransferase in hippocampal tissue. J Neurochem.

[70] Lautié A, Aslanian D, Balkanski M (1978). Non-enzymatic hydrolysis of acetylcholine studied by Raman spectrometry. J Raman Spectrosc.

[71] Aslanian D (1983). Vibrational spectroscopic approach to the study of acetylcholine and related compounds. Life Sci.

[72] Hernández B, Houzé P, Pflüger F (2017). Raman scattering-based multiconformational analysis for probing the structural differences between acetylcholine and acetylthiocholine. J Pharm Biomed Anal.

[73] Tretyn A, Łukasiewicz-Rutkowska H, Kopcewicz J (1997). Isolation, purification and identification of acetylcholine in Pharbitis nil seedlings. Acta Physiol Plant.

[74] Arnaud V, Berthelot M, Evain M (2007). Hydrogen-bond interactions of nicotine and acetylcholine salts: a combined crystallographic, spectroscopic, thermodynamic and theoretical study. Chem A Eur J.

[75] Pawlukojć A, Hetmańczyk Ł (2016). INS, DFT and temperature dependent IR studies on dynamical properties of acetylcholine chloride. Vib Spectrosc.

[76] Bezuglov VV, Gretskaya NM, Esipov SE (2004). Fluorescent-labeled lipophilic analogues of serotonin, dopamine, and acetylcholine: synthesis, mass spectrometry, and biological activity. Russ J Bioorg Chem.

[77] Sayed M, Shinde K, Shah R, Pal H (2016). pH-responsive indicator displacement assay of acetylcholine based on acridine-p-Sulfonatocalix[4]arene supramolecular system: fluorescence off/on switching and reversible pKa shift. ChemistrySelect.

[78] Mangalath S, Abraham S, Joseph J (2017). pH-responsive fluorescence enhancement in graphene oxide-naphthalimide nanoconjugates: a fluorescence turn-on sensor for acetylcholine. Chemistry.

[79] Borden PM, Zhang P, Shivange AV (2020). A Fast genetically encoded fluorescent sensor for faithful in vivo acetylcholine detection in mice, fish, worms and flies. BioRxiv.

[80] Sakai T, Gao Y, Ohno N, Ura N (1991). Novel flow injection method for selective spectrophotometric determination of acetylcholine using thermochromism of ion associates. Chem Lett.

[81] Katz-Brull R, Koudinov AR, Degani H (2005). Direct detection of brain acetylcholine synthesis by magnetic resonance spectroscopy. Brain Res.

[82] Culvenor CCJ, Ham NS (1966). The proton magnetic resonance spectrum and conformation of acetylcholine. Chem Commun (Lond).

[83] Nakahari T, Murakami M, Kataoka T (1989). Shrinkage of rat mandibular acinar cell with acetylcholine detected by video-enhanced contrast microscopy. Jpn J Physiol.

[84] Zhang Y, Bai C, Wang C (1999). Intermolecular forces between acetylcholine and acetylcholinesterases studied with atomic force microscopy. Sci China Ser B-Chem.

[85] Alfonta L, Katz E, Willner I (2000). Sensing of acetylcholine by a tricomponent-enzyme layered electrode using faradaic impedance spectroscopy, cyclic voltammetry, and microgravimetric quartz crystal microbalance transduction methods. Anal Chem.

[86] Yingge Z, Chunli B, Chen W, Delu Z (2001). Force spectroscopy between acetylcholine and single acetylcholinesterase molecules and the effects of inhibitors and reactivators studied by atomic force microscopy. J Pharmacol Exp Ther.

[87] Rosen AD (1992). Magnetic field influence on acetylcholine release at the neuromuscular junction. Am J Physiol.

[88] Correia-de-Sá P, Noronha-Matos JB, Timóteo MA (2013). Bothropstoxin-I reduces evoked acetylcholine release from rat motor nerve terminals: radiochemical and real-time video-microscopy studies. Toxicon.

[89] Hasegawa Y, Kunihara M, Maruyama Y (1982). Determination of picomole amounts of choline and acetylcholine in blood by gas chromatography–mass spectrometry equipped with a newly improved pyrolyzer. J Chromatogr.

[90] Singh AK, Drewes LR (1985). Improved analysis of acetylcholine and choline in canine brain and blood samples by capillary gas chromatography–mass spectrometry. J Chromatogr B Biomed Sci Appl.

[91] Liberato DJ, Yergey AL, Weintraub ST (1986). Separation and quantification of choline and acetylcholine by thermospray liquid chromatography/mass spectrometry. Biomed Environ Mass Spectrom.

[92] Marien MR, Richard JW (1990). Drug effects on the release of endogenous acetylcholine in vivo: measurement by intracerebral dialysis and gas chromatography–mass spectrometry. J Neurochem.

[93] Ikarashi Y, Itoh K, Maruyama Y (1991). Application of FRIT fast atom bombardment liquid chromatography/mass spectrometry for the determination of acetylcholine levels in rat brain regions. Biol Mass Spectrom.

[94] Sakai T, Gao Y-H, Ohno N, Ura N (1991). Batchwise and flow-injection methods for thermo-spectrophotometric determination of acetylcholine and choline with tetrabromophenolphthalein ethyl ester. Anal Chim Acta.

[95] Ishimaru H, Ikarashi Y, Maruyama Y (1993). Use of high-performance liquid chromatography continuous-flow fast atom bombardment mass spectrometry for simultaneous determination of choline and acetylcholine in rodent brain regions. Biol Mass Spectrom.

[96] Acevedo LD, Xu Y, Zhang X (1996). Quantification of acetylcholine in cell culture systems by semi-micro high-performance liquid chromatography and electrospray ionization mass spectrometry. J Mass Spectrom.

[97] Hows MEP, Organ AJ, Murray S (2002). High-performance liquid chromatography/tandem mass spectrometry assay for the rapid high sensitivity measurement of basal acetylcholine from microdialysates. J Neurosci Methods.

[98] Reubsaet JLE, Ahlsen E, Haneborg KG, Ringvold A (2003). Sample preparation and determination of acetylcholine in corneal epithelium cells using liquid chromatography–tandem mass spectrometry. J Chromatogr Sci.

[99] Uutela P, Reinilä R, Piepponen P (2005). Analysis of acetylcholine and choline in microdialysis samples by liquid chromatography/tandem mass spectrometry. Rapid Commun Mass Spectrom.

[100] Lacroix LP, Ceolin L, Zocchi A (2006). Selective dopamine D3 receptor antagonists enhance cortical acetylcholine levels measured with high-performance liquid chromatography/tandem mass spectrometry without anti-cholinesterases. J Neurosci Methods.

[101] Keski-Rahkonen P, Lehtonen M, Ihalainen J (2007). Quantitative determination of acetylcholine in microdialysis samples using liquid chromatography/atmospheric pressure spray ionization mass spectrometry. Rapid Commun Mass Spectrom.

[102] Zhang M-Y, Hughes ZA, Kerns EH (2007). Development of a liquid chromatography/tandem mass spectrometry method for the quantitation of acetylcholine and related neurotransmitters in brain microdialysis samples. J Pharm Biomed Anal.

[103] Korbakov N, Timmerman P, Lidich N (2008). Acetylcholine detection at micromolar concentrations with the use of an artificial receptor-based fluorescence switch. Langmuir.

[104] Wang Y, Wang T, Shi X (2008). Analysis of acetylcholine, choline and butyrobetaine in human liver tissues by hydrophilic interaction liquid chromatography–tandem mass spectrometry. J Pharm Biomed Anal.

[105] Fu B, Gao X, Zhang SP (2008). Quantification of acetylcholine in microdialysate of subcutaneous tissue by hydrophilic interaction chromatography/tandem mass spectrometry. Rapid Commun Mass Spectrom.

[106] Schebb NH, Fischer D, Hein E-M (2008). Fast sample preparation and liquid chromatography–tandem mass spectrometry method for assaying cell lysate acetylcholine. J Chromatogr A.

[107] Prokai L, Fryčák P, Stevens SM, Nguyen V (2008). Measurement of acetylcholine in rat brain microdialysates by LC-isotope dilution tandem MS. Chromatographia.

[108] Jin T (2010). Near-infrared fluorescence detection of acetylcholine in aqueous solution using a complex of rhodamine 800 and p-sulfonatocalix[8]arene. Sensors (Basel).

[109] Persike M, Zimmermann M, Klein J, Karas M (2010). Quantitative determination of acetylcholine and choline in microdialysis samples by MALDI-TOF MS. Anal Chem.

[110] Kirsch SH, Herrmann W, Rabagny Y, Obeid R (2010). Quantification of acetylcholine, choline, betaine, and dimethylglycine in human plasma and urine using stable-isotope dilution ultra performance liquid chromatography–tandem mass spectrometry. J Chromatogr B Anal Technol Biomed Life Sci.

[111] Carrozzo MM, Cannazza G, Pinetti D (2010). Quantitative analysis of acetylcholine in rat brain microdialysates by liquid chromatography coupled with electrospray ionization tandem mass spectrometry. J Neurosci Methods.

[112] Peng L, Jiang T, Rong Z (2011). Surrogate based accurate quantification of endogenous acetylcholine in murine brain by hydrophilic interaction liquid chromatography–tandem mass spectrometry. J Chromatogr B Anal Technol Biomed Life Sci.

[113] Zhang Y, Tingley FD, Tseng E (2011). Development and validation of a sample stabilization strategy and a UPLC-MS/MS method for the simultaneous quantitation of acetylcholine (ACh), histamine (HA), and its metabolites in rat cerebrospinal fluid (CSF). J Chromatogr B Anal Technol Biomed Life Sci.

[114] Liu L, Huang J, Li K (2011). Analysis of acetylcholine from extracellular fluid in brain by in vivo microdialysis and LC-ESI-MS/MS with the stable isotope-labeled internal standard. J Chromatogr B Anal Technol Biomed Life Sci.

[115] Cannazza G, Carrozzo MM, Cazzato AS (2012). Simultaneous measurement of adenosine, dopamine, acetylcholine and 5-hydroxytryptamine in cerebral mice microdialysis samples by LC-ESI-MS/MS. J Pharm Biomed Anal.

[116] Liu C, Shen Y, Yin P (2014). Sensitive detection of acetylcholine based on a novel boronate intramolecular charge transfer fluorescence probe. Anal Biochem.

[117] Wei J, Ren J, Liu J (2014). An eco-friendly, simple, and sensitive fluorescence biosensor for the detection of choline and acetylcholine based on C-dots and the Fenton reaction. Biosens Bioelectron.

[118] Zhang C, Xia Y, Jiang W (2016). Determination of non-neuronal acetylcholine in human peripheral blood mononuclear cells by use of hydrophilic interaction ultra-performance liquid chromatography–tandem mass spectrometry. J Chromatogr B Anal Technol Biomed Life Sci.

[119] Wang Y-H, Kumarihamy M, Wang M (2016). Quantitative determination of betaine, choline, acetylcholine, and 20-hydroxyecdysone simultaneously from atriplex species by UHPLC-UV-MS. Nat Prod Commun.

[120] Martín-Barreiro A, de Marcos S, de la Fuente JM (2018). Gold nanocluster fluorescence as an indicator for optical enzymatic nanobiosensors: choline and acetylcholine determination. Sens Actuators B Chem.

[121] Li L, Zong S, Lu Y (2020). Quaternary-ammonium-modulated surface-enhanced Raman spectroscopy effect: discovery, mechanism, and application for highly sensitive in vitro sensing of acetylcholine. Anal Chem.

[122] Guo J, Wu S, Wang Y, Zhao M (2020). A label-free fluorescence biosensor based on a bifunctional MIL-101(Fe) nanozyme for sensitive detection of choline and acetylcholine at nanomolar level. Sens Actuators B Chem.

[123] Matsuda T, Suzuki Y, Fujisawa T (2020). Imaging mass spectrometry to visualise increased acetylcholine in lungs of asthma model mice. Anal Bioanal Chem.

[124] Helmschrodt C, Becker S, Perl S (2020). Development of a fast liquid chromatography–tandem mass spectrometry method for simultaneous quantification of neurotransmitters in murine microdialysate. Anal Bioanal Chem.

[125] Decheng S, Xia F, Shulin W, Yang L (2021). Analysis of choline, carnitine, acetylcarnitine and acetylcholine in animal feeds, blood and urine using ion chromatography coupled with tandem mass spectrometry. J Chromatogr Sci.

[126] Lamy E, Pilyser L, Paquet C (2021). High-sensitivity quantification of acetylcholine and choline in human cerebrospinal fluid with a validated LC-MS/MS method. Talanta.

[127] Piestansky J, Forgacsova A, Olesova D (2022). Targeted UHPLC-ESI-MS/MS analysis of selected neurotransmitters, tryptophan and its metabolite kynurenine in tau transgenic rat brain tissue: a pivotal study. Separations.

[128] Sakurai T, Iwashita A, Okumura K, et al (2013) Acetylcholine dynamics in cortical networks by an ion image sensor with neurotransmitter-sensitive magnetic nanomachines. In: 2013 transducers and Eurosensors XXVII: the 17th international conference on solid-state sensors, actuators and microsystems (TRANSDUCERS and EUROSENSORS XXVII), pp 760–763

[129] Luo Y, Kim EH, Flask CA, Clark HA (2018). Nanosensors for the chemical imaging of acetylcholine using magnetic resonance imaging. ACS Nano.

[130] Jenden DJ, Choi L, Silverman RW (1974). Acetylcholine turnover estimation in brain by gas chromatography–mass spectrometry. Life Sci.

[131] Lehmann WD, Schulten HR, Schröder N (1978). Determination of choline and acetylcholine in distinct rat brain regions by stable isotope dilution and field desorption mass spectrometry. Biomed Mass Spectrom.

[132] Haug P, Suzuki M, Moritz J (1980). Changes in acetylcholine in caudate nucleus tissue isolated in situ detected by gas chromatography mass spectrometry selected ion monitoring. Biomed Mass Spectrom.

[133] Grumbach ES, Diehl DM, Mazzeo JR (2004). A sensitive ESI-MS HILIC method for the analysis of acetylcholine and choline. LC-GC N Am.

[134] Weintraub ST, Liberato DJ, Yergey AL (1984) Direct determination of acetylcholine in mouse brain by thermospray–mass spectrometry. In: Annual conference on mass spectrometry and allied topics, pp 129–130

[135] Bishop MR, Sastry BV, Stavinoha WB (1977). Identification of acetylcholine and propionylcholine in bull spermatozoa by integrated pyrolysis, gas chromatography and mass spectrometry. Biochim Biophys Acta.

[136] Welsch F, Wenger WC (1980). Acetylcholine in human placenta. Naunyn Schmiedeberg’s Arch Pharmacol.

[137] Hammar C-G, Hanin I, Holmstedt B, Kitz RJ (1968). Identification of acetylcholine in fresh rat brain by combined gas chromatography–mass spectrometry. Nature.

[138] Hariri M (1974). Quantitative measurements of endogenous levels of acetylcholine and choline in tetrathyridia of *Mesocestoides corti *(Cestoda) by means of combined gas chromatograph–mass spectrometry. J Parasitol.

[139] Almalki AH, Alsalahat I, Alharthi MA (2022). Evaluation of greenness of LC-MS chromatographic methods for simultaneous analysis of mixtures of serotonin, dopamine, acetylcholine, GABA and glutamate: AGREE tool application. Separations.

[140] Hanin I, Schuberth J (1974). Labelling of acetylcholine in the brain of mice fed on a diet containing deuterium labelled choline: studies utilizing gas chromatography–mass spectrometry. J Neurochem.

[141] Polak RL, Molenaar PC (1974). Pitfalls in determination of acetylcholine from brain by pyrolysis-gas chromatography/mass spectrometry. J Neurochem.

[142] Murata J, Watanabe T, Sugahara K (2015). High-resolution mass spectrometry for detecting Acetylcholine in Arabidopsis. Plant Signal Behav.

[143] Miller JV, LeBouf RF, Kelly KA (2018). The neuroinflammatory phenotype in a mouse model of gulf war illness is unrelated to brain regional levels of acetylcholine as measured by quantitative HILIC-UPLC-MS/MS. Toxicol Sci.

[144] Igarashi K, Sugiyam Y, Kasuya F (2000). Quantification of acetylcholine in brain microdialysates obtained from rat treated with organophosphorus insecticides using LC/MS. Jpn J Forensic Toxicol.

